# Rapid fractionation of mitochondria from mouse liver and heart reveals *in vivo* metabolite compartmentation

**DOI:** 10.1002/1873-3468.14511

**Published:** 2022-10-27

**Authors:** Fay M. Allen, Ana S. H. Costa, Anja V. Gruszczyk, Georgina R. Bates, Hiran A. Prag, Efterpi Nikitopoulou, Carlo Viscomi, Christian Frezza, Andrew M. James, Michael P. Murphy

**Affiliations:** ^1^ MRC Mitochondrial Biology Unit University of Cambridge UK; ^2^ MRC Cancer Unit University of Cambridge UK; ^3^ Department of Biomedical Sciences University of Padova Italy; ^4^ Department of Medicine University of Cambridge UK

**Keywords:** compartmentation, *in vivo*, ischemia, metabolites, mitochondria, rapid fractionation

## Abstract

The compartmentation and distribution of metabolites between mitochondria and the rest of the cell is a key parameter of cell signalling and pathology. Here, we have developed a rapid fractionation procedure that enables us to take mouse heart and liver from *in vivo* and within ~ 30 s stabilise the distribution of metabolites between mitochondria and the cytosol by rapid cooling, homogenisation and dilution. This is followed by centrifugation of mitochondria through an oil layer to separate mitochondrial and cytosolic fractions for subsequent metabolic analysis. Using this procedure revealed the *in vivo* compartmentation of mitochondrial metabolites and will enable the assessment of the distribution of metabolites between the cytosol and mitochondria during a range of situations *in vivo*.

## Graphical abstract

To assess the distribution of metabolites between mitochondria and the cytosol, we developed a rapid fractionation procedure. We take mouse heart/liver and within ~ 30 s stabilise the distribution of metabolites by rapid cooling, homogenisation and dilution, followed by centrifugation of mitochondria through an oil layer to separate mitochondrial and cytosolic fractions for subsequent metabolic analysis.

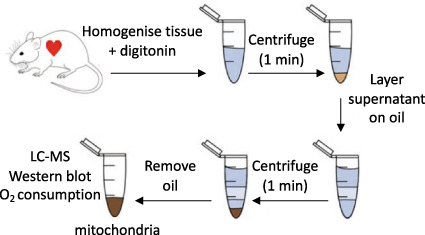

## Abbreviations

**CS**, citrate synthase

**DIC**, dicarboxylate carrier

**DOP**, dioctyl phthalate

**DSMPS**, poly(dimethylsiloxane‐co‐methyl‐phenyl siloxane) oil

**FCCP**, carbonyl cyanide‐*p*‐trifluoromethoxyphenylhydrazone

**GAPDH**, glyceraldehyde‐3‐phosphate dehydrogenase

**GSH**, glutathione

**HILIC**, hydrophobic interaction liquid chromatography

**LC–MS**, liquid chromatography–mass spectrometry

**NEM**, *N*‐ethylmaleimide

**P5P**, pyridoxal‐5‐phosphate

**PDH**, pyruvate dehydrogenase

**SSA**, 5‐sulfosalicylic acid

**STE**, sucrose‐Tris‐EGTA buffer

**TCA**, tricarboxylic acid

**WI**, warm ischemia

Changes in the distribution of metabolites between mitochondria and the rest of the cell are a key aspect of cell function and signalling [1, 2, 3]. For example, citrate moves from the mitochondria to the cytosol to generate acetylCoA, which can be used to make malonylCoA for fatty acid synthesis, or to acetylate histone lysine residues in the nucleus [4]. Succinate is another metabolite that is thought to move from the mitochondria to the cytosol where it controls the activity of prolyl hydroxylases involved in HIF‐1α stabilisation, and to the nucleus, in order to regulate the activity of 2‐oxoglutarate‐dependent dioxygenases involved in DNA and histone demethylation [5].

Exploring metabolite distribution *in vivo* necessitates isolating mitochondria from the cytosol of tissue samples. Importantly, during this process, the distribution of metabolites between and within the mitochondria and cytosol must be stabilised very rapidly, as otherwise enzyme activity and transport across the mitochondrial inner membrane will disrupt *in vivo* metabolite levels. Here we developed procedures for the rapid stabilisation of metabolite levels in the mitochondria and cytosol of mouse heart and liver, followed by their separation. As a proof of concept, we used this procedure to assess how metabolite distribution altered during warm ischemia, as occurs during heart attack, stroke and organ transplantation. These approaches will be of use in exploring mitochondrial metabolite compartmentation in health and disease.

## Materials and methods

### Animals

Rat tissue was harvested from female Wistar rats (Charles River Laboratories, Margate, Kent, UK) at 8–12 weeks of age. Mouse tissue was harvested from female C57BL/6 mice (Charles River Laboratories) at 6–8 weeks of age. Animals were kept on 12 h light/dark cycles in specific pathogen‐free animal facilities with *ad libitum* access to food and water. Animals were culled within the same 3 h window of the day. All animal experiments were approved by the UK Home Office under the Animals (Scientific Procedures) Act 1986 and the University of Cambridge Animal Welfare Policy. For rapid mitochondrial extraction experiments, mice were culled by cervical dislocation.

### Heart and liver warm ischemia

Liver and heart were rapidly extracted (30–40 s) from female C57/Bl6J mice and placed into 2‐mL Eppendorf tubes pre‐warmed in a heat block at 37 °C containing 500 μL PBS (137 mm NaCl, 2.7 mm KCl, 10 mm Na_2_HPO_4_, 1.8 mm KH_2_PO_4_, pH 7.4), and a cut‐off pipette tip was used to suspend the tissue above the liquid so as to maintain humidity without leaching metabolites. Tubes were incubated at 37 °C for 30 min before the organ was removed and mitochondria isolated according to the rapid protocols described below.

### Conventional mitochondrial isolations

To isolate rat liver mitochondria, the rat was killed by stunning and cervical dislocation and the liver was removed and homogenised in ice‐cold STE buffer (250 mm sucrose, 5 mm Tris, 1 mm EGTA, pH 7.4) using a Potter‐Elvehjem tissue grinder (Wheaton, Palmer, MA, USA). The homogenate was centrifuged at 1000 ***g*** for 3 min at 4 °C and the supernatant was centrifuged at 10 000 ***g*** for 10 min at 4 °C to pellet mitochondria. The mitochondrial pellet was resuspended in STE and centrifuged at 10 000 ***g*** for 10 min at 4 °C. The final mitochondrial pellet was suspended in 5 mL STE.

To isolate rat heart mitochondria, the hearts were homogenised in ice‐cold STEB buffer (STE buffer supplemented with 0.1% (w/v) fatty acid‐free BSA) using a 55 mL Potter‐Elvehjem tissue grinder (Wheaton). The homogenate was centrifuged at 1000 ***g*** for 5 min at 4 °C and the supernatant was filtered through two layers of pre‐wetted muslin and centrifuged at 10 000 ***g*** for 10 min at 4 °C to pellet mitochondria. The mitochondria were resuspended and centrifuged at 10 000 ***g*** for 10 min at 4 °C, and the final mitochondrial pellet was resuspended in 400 μL STE buffer.

### Rapid isolation of mouse heart mitochondria

Just before culling the mouse, 4 × 1.5 mL Eppendorf tubes were prepared by layering 300 μL of Heart Oil Mix (38 : 62 silicone oil : dioctyl phthalate; density: 1.0158 g·mL^−1^ at RT), above 100 μL of either 15% glycerol (density: 1.0390 g·mL^−1^ at RT; for WB), 15% formic acid (density: 1.033 g·mL^−1^; for liquid chromatography–mass spectrometry (LC–MS)) or heavy Seahorse assay buffer (HSAB, density: 1.049 g·mL^−1^; for Seahorse respirometry). The silicone oil was poly(methylphenylsiloxane) oil viscosity 450‐550 cSt (Sigma #378496). These tubes were kept on ice in a metal block until required. It was very important that the tubes were prepared just before culling as the components in the oil layer can separate with time leading to inversion of the three layers upon centrifugation. It is also essential that the oil mixture be thoroughly vortexed just prior to preparing the tubes. The heart was then rapidly extracted (within 20–25 s of culling the animal) from a female C57/Bl6J mouse and placed into cold ‘Heart Homogenisation Buffer’ (25 mm sucrose, 5 mm Tris, 1 mm EGTA, 87.5 mm ammonium bicarbonate, 100 μg·mL^−1^ digitonin, pH 7.4; density: 1.0075 g·mL^−1^ at RT) to remove excess blood. The heart was rapidly chopped with scissors into a 7‐mL glass tissue grinder (Kimble Chase, Rockwood, TN, USA; 885300‐0007) containing 3 mL ‘Heart Homogenisation Buffer’, pre‐cooled on ice. All subsequent procedures were performed on ice. The tissue was homogenised by three strokes using pestle A (loose fitting) and three strokes of pestle B (tight fitting). A fraction of this homogenate was retained for analysis and termed ‘Homogenate’. Then 1‐mL aliquots of the homogenate were placed in 2‐mL Eppendorf tubes and four of these tubes were centrifuged in parallel at 1000 ***g*** for 1 min at 4 °C to pellet cell debris. A fraction of this supernatant was retained for analysis and termed ‘Supernatant’; 650 μL of this supernatant was carefully layered on to the Heart Oil Mix in the 4 1.5‐mL Eppendorf tubes that were prepared in advance. These were centrifuged for 1 min at 9727 ***g*** at 4 °C in a swing‐out rotor (Heraeus, Hanau, Germany; #75007592) fitted in a Heraeus Biofuge Primo R centrifuge (Thermo Scientific, Loughborough, UK). It is important to use a swing‐out rotor for this step, so that the mitochondria pass directly into the lower acid layer without smearing on the sides of the tube. The layer above the oil, termed the ‘Cytosolic’ layer, and the majority of the oil were aspirated off and the tip of the tube was placed in a dry ice and ethanol bath. Once the lower layer had frozen solid, the remnants of the unfrozen oil layer were washed off using hexane that had been cooled on dry ice. The four mitochondrial pellets were then pooled to obtain the final mitochondrial pellet.

### Rapid isolation of mouse liver mitochondria

Just before culling the animal, 4 × 1.5 mL Eppendorf tubes were prepared by layering 300 μL Liver Oil Mix (60 : 40 silicone oil:dioctyl phthalate; density: 1.066 g·mL^−1^ at RT), above 100 μL of either: 23% glycerol (density: 1.0598 g·mL^−1^ at RT; for WB), 25% formic acid (density: 1.055 g·mL^−1^; for LC–MS) or heavy Seahorse assay buffer (HSAB, density: 1.049 g·mL^−1^; for Seahorse). These tubes were kept on ice in a metal block until required. It was very important that the tubes were prepared just before culling as the components in the oil layer can separate with time leading to inversion of the three layers upon centrifugation. It is also essential that the oil mixture be thoroughly vortexed just prior to preparing the tubes. The liver was then rapidly extracted from a female C57/Bl6J mouse (within 30–35 s of culling the animal) and placed into ice‐cold ‘Liver Homogenisation Buffer’ (200 mm sucrose, 5 mm Tris, 1 mm EGTA, 100 μg·mL^−1^ digitonin, pH 7.4; density: 1.0248 g·mL^−1^ at RT) to remove excess blood. The whole liver was then placed in a 7‐mL glass tissue grinder (Kimble Chase; 885300‐0007) containing 5 mL ‘Liver Homogenisation Buffer’ pre‐cooled on ice to 4 °C. From this point onwards, the whole experiment was performed on ice. The tissue was homogenised by three strokes using pestle A (loose fitting) followed by three strokes of pestle B (tight fitting). A fraction of this homogenate was retained for analysis and termed ‘Homogenate’. Then 1‐mL aliquots of the homogenate were placed in 2‐mL Eppendorf tubes and four of these tubes were centrifuged in parallel at 1000 ***g*** for 1 min at 4 °C to pellet cell debris. A fraction of this supernatant was retained for analysis and termed ‘Supernatant’. Four aliquots of 200 μL of the resulting supernatant were mixed with ice‐cold 500 μL ‘Spin Buffer’ (150 mm sucrose, 5 mm Tris, 1 mm EGTA, 25 mm ammonium bicarbonate, pH 7.4; density: 1.0200 g·mL^−1^ at RT) to reduce the density of the suspension; 700 μL of each of these four aliquots were carefully layered above the oil in the 4 1.5‐mL Eppendorf tubes that were prepared in advance. These were centrifuged for 1 min at 9727 ***g*** at 4 °C in a swing‐out rotor (Heraeus; #75007592) fitted in a Heraeus Biofuge Primo R centrifuge (Thermo Scientific). It is important to use a swing‐out rotor for this step, so that the mitochondria pass directly into the lower acid layer without smearing on the sides of the tube. The layer above the oil, termed the ‘Cytosolic’ layer, and the majority of the oil were aspirated off and the tip of the tube was placed in a dry ice and ethanol bath. Once the lower layer had frozen solid, the remaining oil (which does not freeze solid) was washed off using dry ice‐cold hexane. The four mitochondrial pellets were then pooled to obtain the final mitochondrial pellet.

### Western blotting

Samples were heated in an appropriate volume of 4× Laemmli sample buffer (125 mm Tris (pH 6.8), 4% (w/v) SDS, 40% (v/v) glycerol, 25 mg·mL^−1^ Bromophenol Blue and freshly added β‐Mercaptoethanol (BME, 5% (v/v))) for 5 min at 95 °C. Then 10 μg protein was loaded per lane of a mini‐PROTEAN® TGXTM Precast 4–20% gradient gel (Bio‐Rad, Watford, UK). Proteins were electrophoresed at 100 V until the loading dye ran off the gel in running buffer (25 mm Tris (pH 8.3), 192 mm glycine and 0.1% (w/v) SDS). Precision Plus Protein Dual Color standards (Bio‐Rad) or MagicMark™ XP Western Protein Standard (ThermoFisher) was loaded on all gels. Electrophoresed proteins were transferred onto PVDF membranes using either a wet or semi‐dry transfer method. For wet transfer, the proteins were transferred to Immobilon®‐FL PVDF membranes (Merck Millipore, Poole, UK: IPFL00010) for 1 h at 100 V, 4 °C. The PVDF membranes were activated in methanol for 15 s before pre‐equilibration of the membranes, gels, filter papers and fibre pads in pre‐chilled transfer buffer (25 mm Tris, 192 mm glycine, 20% (v/v) methanol, pH 8.4 (unadjusted) at 4 °C). For semi‐dry transfer, the membranes were transferred using Trans‐Blot® Turbo™ Transfer packs (Bio‐Rad: 1704157) at 2.5 A (constant), 25 V (max) for 7 min. Post‐transfer, the membranes were blocked for 1 h at RT in Odyssey® Blocking Buffer (PBS) (LI‐COR Biosciences, Cambridge, UK: 927‐40000) with gentle shaking (Bibby Stuart Platform Rocker STR6 at 30 r.p.m.). The membranes were then incubated with primary antibodies overnight at 4 °C in 4% (v/v) blocking buffer in PBST (137 mm NaCl, 2.7 mm KCl, 10 mm Na_2_HPO_4_, 1.8 mm KH_2_PO_4_ and 0.05% (v/v) Tween‐20). The membranes were incubated in secondary antibodies in 4% (v/v) blocking buffer/PBST for 1 h at RT with shaking, protected from light. Primary antibodies used were: rabbit anti‐GAPDH (1 : 10 000; Sigma, Gillingham, UK, G9545), mouse anti‐PDH (1 : 1000; Abcam, Cambridge, UK, ab110333) and mouse anti‐NDUFB8 (1 : 2000; Abcam, ab110242). Secondary antibodies: goat anti‐mouse (1 : 10 000; IRDye® 680RD goat anti‐mouse IgG (H+L): (LI‐COR Biosciences: 926‐68070) 1 : 10000) and goat anti‐rabbit (1 : 20000; IRDye® 800CW goat anti‐rabbit IgG (H+L): (LI‐COR Biosciences: 926‐32211) 1 : 20 000). Excess primary and secondary antibodies were removed from the membrane by washing in PBST (four buffer changes in 1 h) with gentle shaking (30 r.p.m.). Final washes were performed in the absence of Tween‐20 (PBS only). The signal intensities of the target bands were measured as fluorescence emission at 680 or 800 nm (depending on the secondary antibody used) with the Odyssey® CLx Infrared Imaging System (LI‐COR Biosciences). The signal intensities of target bands were normalised against control bands using image studio™ lite software (LI‐COR Biosciences).

### Seahorse analysis of isolated mitochondria

The method was adapted from [6]. An XF96 cartridge was hydrated with 200 μL of calibrant solution and incubated at 37 °C overnight. Liver and heart mitochondria were isolated as described above, using a lower layer of ‘Heavy Mitochondrial Assay Solution’ (HMAS): 700 mm sucrose, 220 mm mannitol, 10 mm KH_2_PO_4_, 5 mm MgCl_2_, 2 mm HEPES, 1 mm EGTA, 0.2% (w/v) fatty acid‐free BSA and pH 7.2. The pellets from four tubes were combined and protein concentration was assessed by bicinchoninic acid (BCA) assay. Pre‐warmed MAS sucrose (300 mm), mannitol (220 mm), KH_2_PO_4_ (10 mm), MgCl_2_ (5 mm), HEPES (2 mm), EGTA (1 mm), 0.2% (w/v) fatty acid‐free BSA, pH 7.2 and density 1.015 g·mL^−1^ were supplemented with 4 μg·mL^−1^ rotenone and 5 mm succinate (MAS + R + S). Carbonyl cyanide‐*p*‐trifluoromethoxyphenylhydrazone (FCCP) was added as a final concentration 4 μm and antimycin A at a final concentration 3 μm. Cartridge was placed into Seahorse and calibrated. Mitochondria were diluted to 100 μg protein·mL^−1^ in cold MAS + R + S and 2 μg protein was plated onto each well of a XF96 cell culture microplate. The plate was centrifuged for 20 min at 2000 ***g*** and wells were made up to 180 μL with pre‐warmed MAS + R + S and then analysed.

### Glutathione recycling assay

The glutathione (GSH) recycling assay was used to measure total GSH equivalents (= GSH + 2 × GSSG) [7]. Mitochondrial samples for analysis following rapid fractionation were obtained by using either 100 μL of 15% (w/v) 5‐sulfosalicylic acid (SSA; density: 1.07995 g·mL^−1^) or 15% (heart) or 23% (liver) glycerol as the bottom layer (glycerol was used when WB analysis was run in parallel). Isolated mitochondria from duplicate tubes were combined and pellets were resuspended. For 15% SSA samples, 400 μL of H_2_O was added to dilute SSA to 5% (w/v). For glycerol samples, 40 μL of mitochondria was added to 40 μL of 10% SSA to achieve a final concentration of 5% SSA. Samples were vortexed and then centrifuged at 16 000 ***g*** for 10 min at 4 °C, and 10 μL of supernatant was analysed in triplicate on a 96‐well plate. A standard curve of 0, 5, 10, 25, 50 and 100 μm GSH was made in 5% (w/v) SSA. Samples were incubated in 0.5 mm NADPH, 0.5 mm 5,5′‐dithio‐bis‐[2‐nitrobenzoic acid] (DTNB) and 4 U·mL^−1^ glutathione reductase (GR) from Baker's yeast in 143 mm sodium phosphate, 6.3 mm EDTA, pH 7.5. Production of 2‐nitro‐5‐thiobenzoic acid (TNB) was followed by measuring absorbance at 412 nm using a 96‐well plate kinetic spectrophotometer (Molecular Devices, San Jose, CA, USA) for 10 min at 25 °C. Rates of samples were compared to standard curve to determine the concentration of GSH equivalents in the sample.

### Citrate synthase assay

The method was adapted from [8] for a 96‐well plate format. To each well was added 80 μL assay buffer (100 μm DTNB and 300 μm acetylCoA in 25 mm KH_2_PO_4_) along with sample (8 μg protein in 100 μL KPi buffer), and the reaction was started by the addition of 20 μL of 500 μm oxaloacetate. Absorbance at 412 nm was measured for 10 min at 30 °C. ε_412_ = 13 600 m ^−1^·cm^−1^. Background activity was measured by replacing sample with KPi buffer and with a no‐oxaloacetate control.

### Liquid chromatography–mass spectrometry

For LC–MS analysis, samples were treated as follows: for homogenate, supernatant and cytosolic fractions 40 μL were added to 400 μL 25% (liver) or 15% (heart) formic acid. Mitochondrial samples obtained by centrifugation through oil into either 25% (liver) or 18% (heart) formic acid were vortexed. All samples were then centrifuged (10 min at 17 000 ***g***). Supernatants of quadruplicate mitochondrial samples were pooled, then all samples were dried under vacuum (miVac Quattro concentrator; Genevac, Ipswich, UK) for 4 h, with a temperature capped of 40 °C. Dried samples were stored at −80 °C until just before LC–MS analysis when they were resuspended in 75 μL H_2_O, and agitated for 10 min at 4 °C to resuspend metabolites and then centrifuged for 1 min at 17 000 ***g***, 4 °C. The supernatants were transferred to high recovery vials (9512S‐3MP‐RS or Low adsorption vials, 29659‐U; Sigma) and stored at −80 °C until LC–MS analysis.

LC–MS analyses were performed on a Q Exactive Orbitrap (Thermo Fisher Scientific, Bremen, Germany) mass spectrometer coupled to an Ultimate 3000 RSLC system (Dionex). The liquid chromatography system was fitted with either a ZIC‐HILIC column (150 mm × 4.6 mm) or a ZIC‐pHILIC column (150 mm × 2.1 mm) guard columns (20 mm × 2.1 mm) (all Merck, Darmstadt, Germany). The ZIC‐HILIC column mobile phase was: 0.1% formic acid in water (aqueous) and 0.1% formic acid in acetonitrile (organic). The ZIC‐pHILIC column mobile phase was: 20 mm ammonium carbonate + 0.1% ammonium hydroxide (aqueous) and acetonitrile (organic). The mass spectrometer was operated in full MS and polarity switching mode. Samples were randomised in order to avoid bias due to machine drift and processed blindly. The acquired spectra were analysed using xcalibur qual browser and xcalibur quan browser software (Thermo Fisher Scientific, Bremen, Germany) by referencing to an internal library of compounds.

### Statistical analysis

Data analysis was performed with graphpad prism 7.0 (GraphPad Software, San Diego, CA, USA) and the statistical tests used are indicated in the figure legends. Results were expressed as mean ± standard error of the mean (SEM).

## Results and Discussion

### Rapid separation of mitochondria from the cytosol in mouse heart and liver

**Fig. 1:**
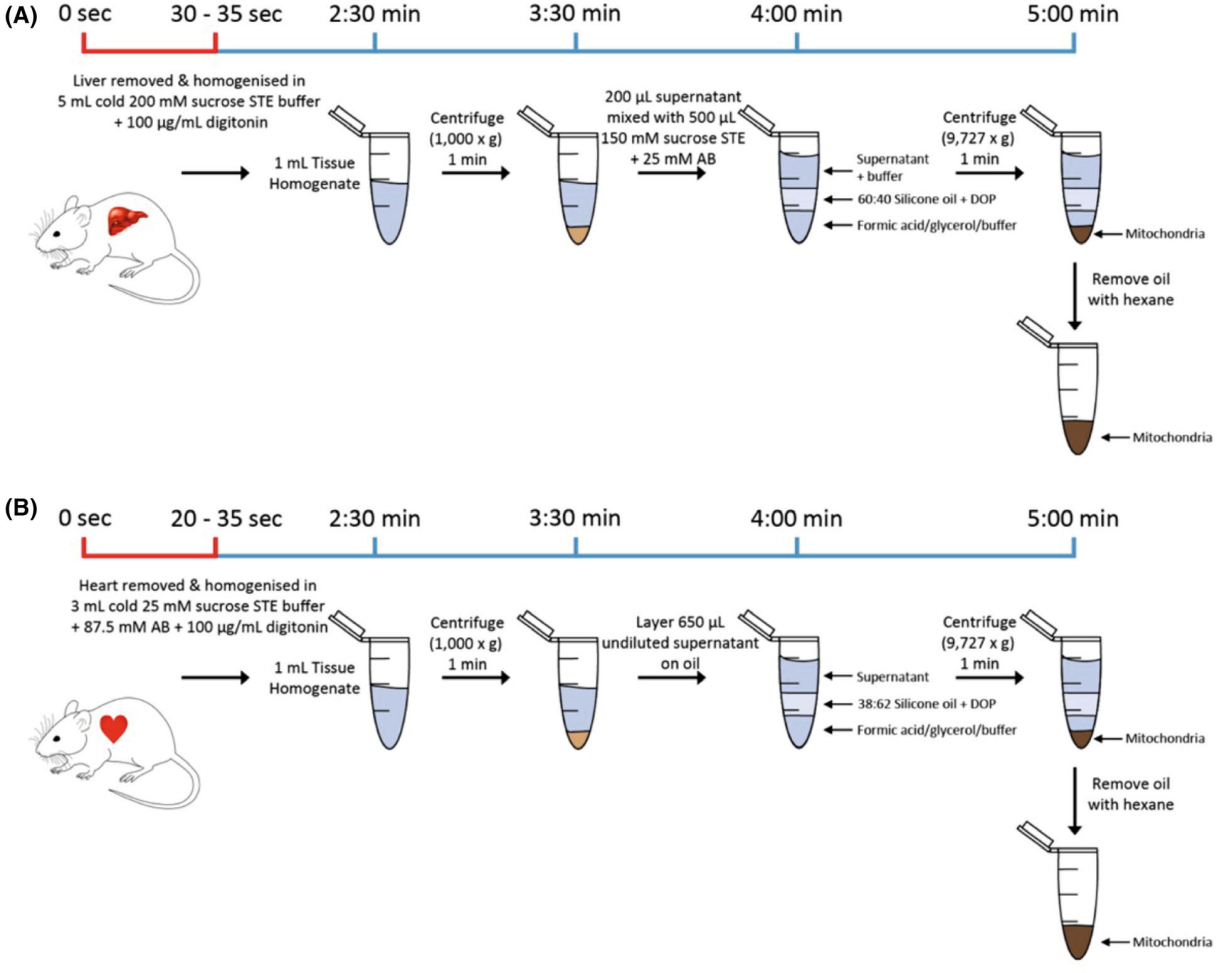
Rapid tissue fractionation procedures for mouse liver and heart. (A) The liver is removed and homogenised in 5 mL of Liver homogenisation Buffer (200 mm sucrose, 5 mm Tris–HCl, 1 mm EGTA, pH 7.4, supplemented with 100 μg·mL^−1^ digitonin). 1 mL portions are centrifuged (1000 ***g*** for 1 min), and 200 μL supernatant is mixed with 500 μL Spin Buffer (150 mm sucrose, 5 mm Tris–HCl, 1 mm EGTA, 25 mm ammonium bicarbonate pH 7.4). Seven hundred microliter of this mixture is layered onto 300 μL of oil (60 : 40 (v/v) poly(dimethylsiloxane‐co‐methyl‐phenyl siloxane) oil (DSMPS)/dioctyl phthalate (DOP)) and centrifuged for 1 min at 9727 ***g***. For LC–MS analysis, the lower layer was 23% formic acid (FA), and for protein analysis, it was 23% glycerol. (B) The heart is removed and homogenised in 3 mL of Heart homogenisation Buffer (25 mm sucrose, 87.5 mm ammonium bicarbonate, 5 mm Tris–HCl, 1 mm EGTA, pH 7.4 supplemented with 100 μg·mL^−1^ digitonin. One milliliter portions were centrifuged at 1000 ***g*** for 1 min and 650 μL of this supernatant was layered directly onto 300 μL of oil (38 : 62 (v/v) DSMPS : DOP) and centrifuged for 1 min at 9727 ***g***. For LC–MS analysis, the lower layer was 15% FA, and for protein analysis, it was 15% glycerol. The timeline starts at the point of cervical dislocation with it taking 20–35 s to achieve organ removal and homogenisation in ice‐cold buffer.

To accurately assess mitochondrial and cytosolic metabolite pools within tissues *in vivo*, we must both stop metabolic reactions and mitochondrial metabolite transport, while also separating mitochondria from the cytosolic fraction. The procedures we developed to do this for mouse liver and heart are shown in Fig. 1A,B, respectively. We simultaneously cooled, homogenised and diluted tissues in the presence of the detergent digitonin. The cold tissue homogenate was generated within ~ 30 s of killing the mouse. Note that achieving such rapid tissue homogenisation requires two people to work together, with one culling the mouse and retrieve the organ who then passes it to the other person to homogenise immediately. Dilution should slow metabolite transport across the mitochondrial inner membrane, as most mitochondrial carriers are metabolite exchangers [9, 10]. Rapid cooling will also slow these mitochondrial carriers as well as enzymatic degradation of metabolites in the cytosol. Digitonin selectively disrupts cholesterol‐containing membranes, including the mitochondrial outer membrane leaving the cholesterol‐free mitochondrial inner membrane intact [11]. The homogenate was then centrifuged briefly to remove nuclei and debris, leaving the cytosol and mitochondria in the supernatant. To separate mitochondria from the cytosol, the supernatant was placed over a layer of oil and then centrifuged so that the dense mitochondria passed through the oil into acid, leaving the cytosol above the oil.

**Fig. 2:**
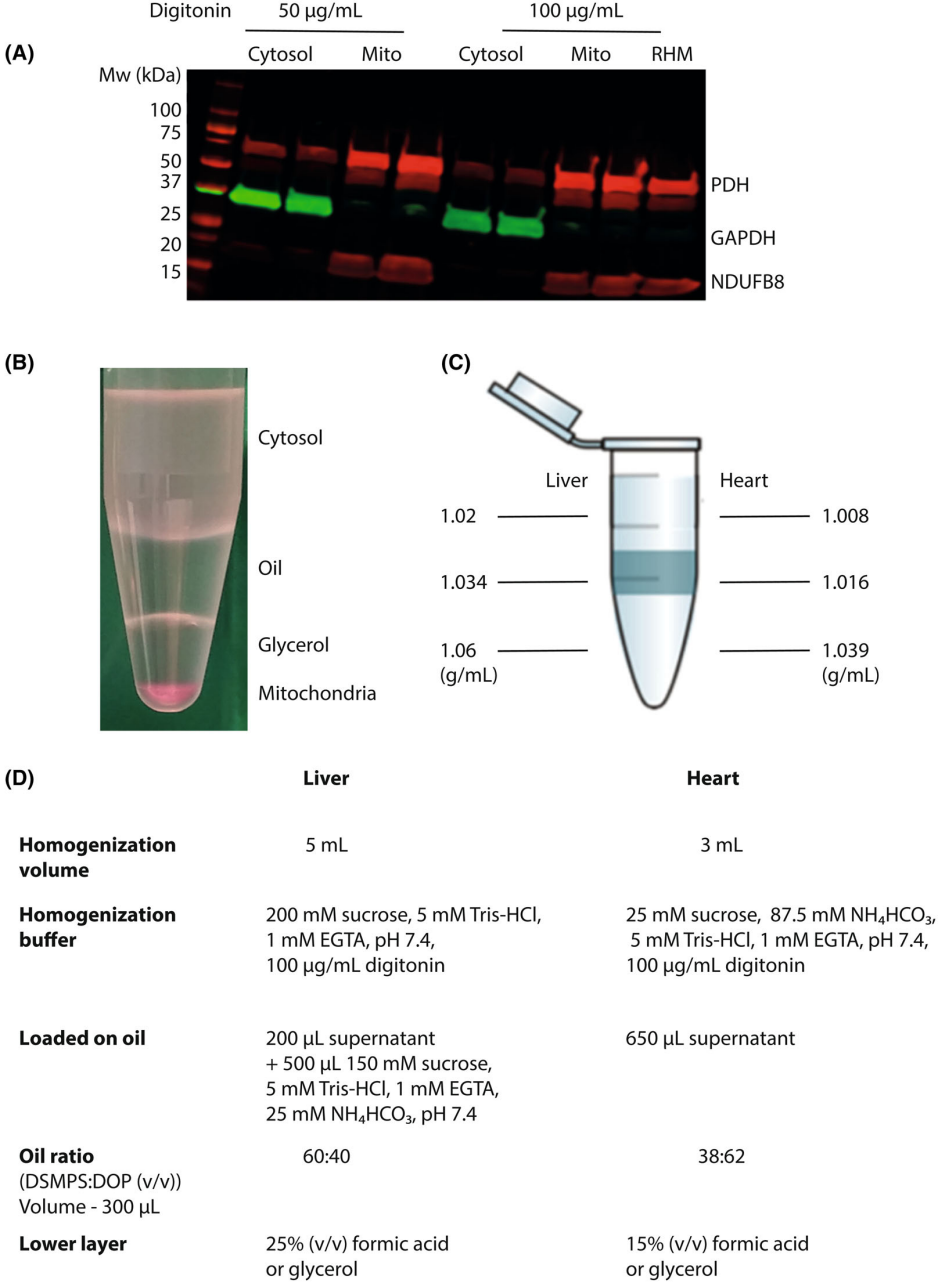
Optimisation of tissue fractionation. (A) Typical experiment to optimise the digitonin concentration in rat heart. A rat heart was homogenised using the indicated concentrations of digitonin and the cytosolic and mitochondrial fractions separated as shown in Fig. 1B. The fractions were analysed by immunoblotting using Protein Plus Dual Color standard and the following primary antibodies: anti‐GAPDH, rabbit, 1 in 10 000; anti‐PDH mouse, 1 in 2000; anti‐NDUFB8 mouse, 1 in 2000. This is compared with rat heart mitochondria isolated by conventional differential centrifugation (RHM). (B) Illustration of a typical separation of mouse heart mitochondria from cytosol by centrifugation as described in Fig. 1B. For this experiment, tetramethylrhodamine (3 μm) was added to the homogenate to facilitate visualisation of the mitochondrial pellet. (C) The optimised densities at room temperature for the phases used in fractionating mouse liver and heart by centrifugation through oil. (D) Optimised compositions for the buffers and phases used in homogenising and fractionating mouse liver and heart.

Procedures were initially optimised using rat liver and heart, due to the higher amounts of tissue available, and then extended to whole mouse hearts and livers. An extensive range of experiments were carried out to optimise digitonin concentrations, buffer compositions, silicone oil densities, and the composition of the lower acid/glycerol layers. In these, the efficacy of separation of the mitochondrial pellet from the cytosolic fraction was routinely assessed by immunoblotting for the distribution of the cytosolic marker protein glyceraldehyde‐3‐phosphate dehydrogenase (GAPDH), the mitochondrial matrix protein pyruvate dehydrogenase (PDH) and the mitochondrial inner membrane protein NDUFB8, which is a component of complex I. A typical optimisation experiment from rat heart showing good separation of the mitochondrial and cytosolic compartments at various concentrations of digitonin is shown in Fig. 2A. Another important aspect to the optimisation procedure was to get ‘clean’ separation of the fractions with no accumulation of material at the oil/buffer or oil/lower layer interfaces. Visualisation of this was aided by spiking homogenates with the dye tetramethylrhodamine, which binds to mitochondrial membranes staining them pink. A typical separation of mitochondria from the cytosol is shown in Fig. 2B, where it can be seen that the mitochondrial pellet is separated from the cytosolic fraction with no material trapped at the liquid interfaces. These experiments led to the finalised densities at room temperature for the buffer and the oil layers for mouse heart and liver Fig. 2C. The compositions of the buffer, oil layer and the lower layer below the oil are shown in Fig. 2D.

The optimised procedures are described in detail in the Materials and methods section. To summarise, the mouse is killed by cervical dislocation and the whole liver or heart removed rapidly, transferred to ice‐cold buffer and homogenised. The homogenates are briefly centrifuged to remove nuclei and unbroken tissue and the supernatants removed. Aliquots are then loaded onto an oil layer for separation of the cytosolic and mitochondrial fractions by centrifugation through oil. The layer below the oil can, for example, be formic acid to denature the mitochondria, precipitate protein and leave an acidic extract for mass spectrometric analysis of metabolites. Alternatively, this layer can be glycerol for mitochondrial protein analysis or sucrose buffer when functional mitochondria are required. After centrifugation, the cytosolic upper layer can be removed for analysis. It is important to remove all traces of oil before LC–MS analysis. To do this, after centrifugation, the cytosolic layer is removed, and then most of the oil is carefully removed using a pipette and then the tube is frozen on dry ice. Then cold hexane is used to wash residual oil off the frozen pellet, before further evaporation leaves a clean acid extract for LC–MS analysis.

### Quantification and quality assessment of mitochondrial recovery from tissues

**Fig. 3:**
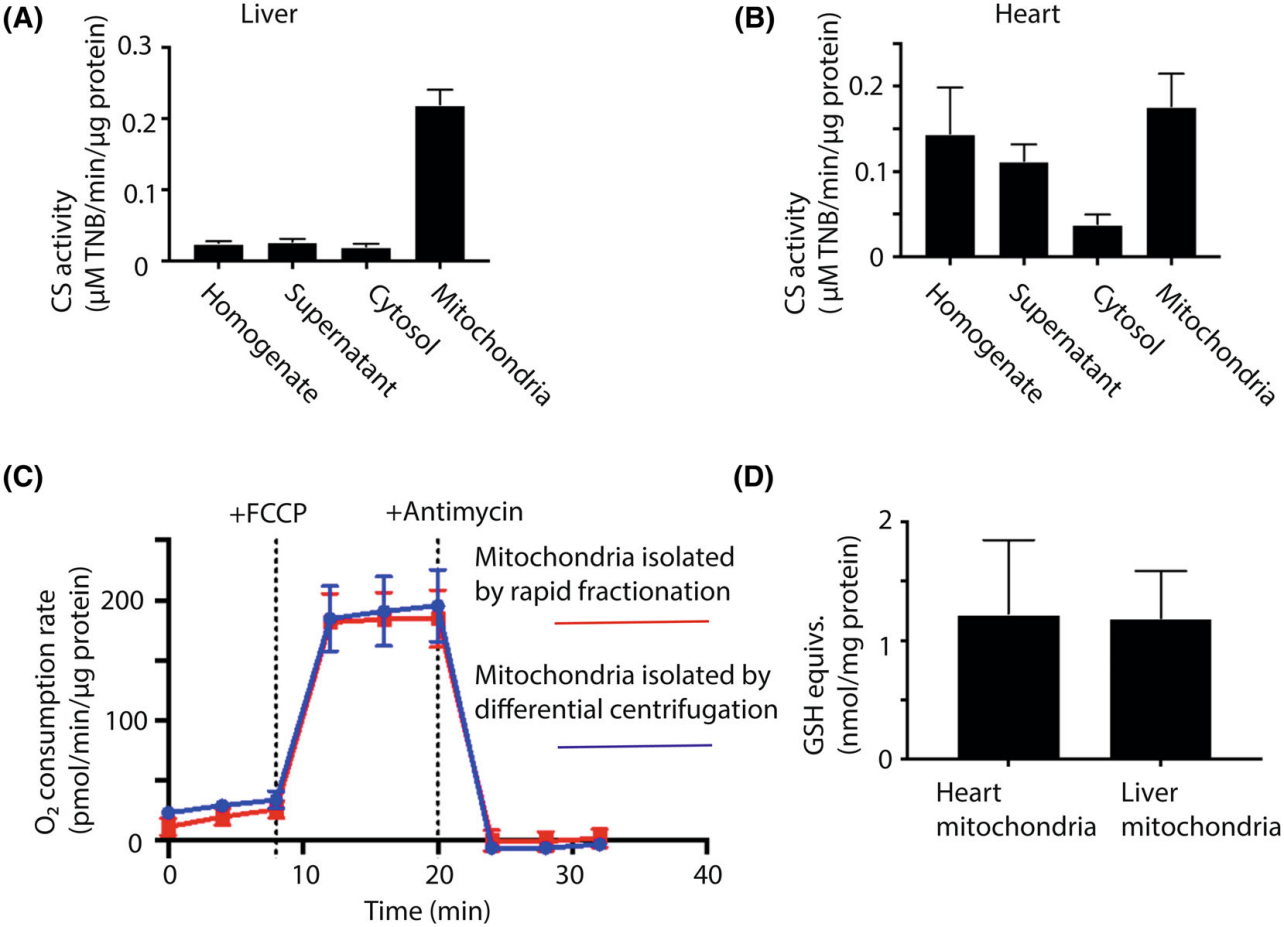
Analysis of mitochondrial enrichment following tissue fractionation. (A, B) During the fractionation of mouse liver or heart tissues by the procedures shown in Fig. 1, samples were taken at all stages and the volume, protein concentration and CS specific activities were determined for the liver (A) and heart (B). Data are means ± SEM, *n* = 4 (liver) or *n* = 3 (heart). (C) Mouse liver mitochondria were isolated by conventional homogenisation followed by differential centrifugation, or by rapid fractionation. For rapid fractionation, the procedure was as described in Fig. 1A, except that the lower layer below the oil was replaced with 100 μL 300 mm sucrose, 220 mm mannitol, 10 mm KH_2_PO_4_, 5 mm MgCl_2_, 2 mm HEPES, 1 mm EGTA, 0.2% (w/v) fatty acid‐free BSA, pH 7.2. The mitochondrial pellet from rapid fractionation was resuspended in the same buffer. The mitochondria from both isolation procedures were then analysed using a Seahorse respirometer in the presence of succinate (5 mm) with additions of FCCP (4 μm) and antimycin (3 μm) where indicated. Data are typical traces showing means ± SEM of four technical replicates. (D) Mouse heart and liver mitochondria were isolated *via* the rapid procedure described in Fig. 1. After resuspension of the mitochondrial pellets in glycerol, the amounts of total GSH equivalents were determined by the GSH recycling assay and the protein concentration was determined in parallel. Data are means ± SEM, *n* = 3.

To quantify the recovery of mitochondria from the rapid fractionation of mouse heart and liver, we measured the activity of the mitochondrial matrix marker enzyme citrate synthase (CS) and the protein concentration in all fractions throughout the isolation procedure (Fig. 3A,B). This showed that CS activity was significantly enriched in the mitochondrial fraction compared to that of the cytosol (~ 12‐fold in liver, ~ 6‐fold in the heart). This is consistent with intact mitochondria being centrifuged through the oil layer into the lower fraction with minimal mitochondrial rupture, which would enhance the CS activity in the cytosolic fraction. For mouse liver, typically ~ 80–90% of the CS activity in the initial tissue homogenate was recovered in the mitochondrial fraction, while in the heart recovery was ~ 40–50%, presumably due to lower release of mitochondria from the more fibrous heart tissue during homogenisation. There was similar, low contamination of the rapidly and conventionally isolated liver and heart mitochondria as judged by immunoblotting for marker proteins of lysosomes, peroxisomes, endoplasmic reticulum and nuclear membrane fragments (Fig. S1). This finding suggests that the metabolites assessed in the mitochondrial fraction are dominated by those present within mitochondria and are not greatly affected by contamination. One exception was that more peroxisomes were present in the rapidly isolated heart mitochondria than in those isolated by conventional procedures. The reason for this is unclear, but may reflect the use of digitonin in the rapid isolation. Adapting the buffer composition below the oil layer enabled us to isolate respiratory competent mitochondria, allowing measurement of respiration by Seahorse respirometry (Fig. 3C). The respiratory activity of mitochondria isolated by centrifugation through oil was similar to that of mitochondria isolated by conventional differential centrifugation (Fig. 3C). Importantly, the stimulation of respiration upon uncoupling by addition of FCCP was similar for both mitochondrial preparations, indicating that the mitochondria are well coupled. Next, we measured the combined content of glutathione (GSH) and glutathione disulphide (GSSG) in the mitochondrial fraction, expressed relative to the protein content (Fig. 3D). These were similar to literature values for intact isolated mitochondria [7]. Furthermore, permeabilisation of the mitochondrial inner membrane by addition of alamethicin (40 μg·mL^−1^) to mouse liver supernatants prevented the accumulation of GSH and GSSG in the mitochondrial fraction below the oil. Together these findings indicate that centrifugation through oil leads to the rapid isolation of intact, functional mitochondria that retain their matrix metabolite contents.

### LC–MS analysis of metabolites from tissue fractions

To assess the method and metabolism in liver and heart tissues, we focussed on carrier‐transported polar metabolites, such as those in the TCA cycle, amino acids, glycolytic intermediates and metabolic cofactors. For this, we analysed the samples by LC–MS using hydrophobic interaction liquid chromatography (HILIC). To enhance metabolite coverage, we analysed samples in parallel using two separate columns, a ZIC‐HILIC column at a lower pH gradient range (2.8–8) and a ZIC‐pHILIC column at a high pH gradient range (9.2–10). Using both columns enabled us to detect 92 distinct metabolites in the heart (Tables S1 and S2) and 115 in the liver (Tables S3 and S4).

The raw ion intensities for the metabolites detected in the homogenates, supernatants, cytosolic and mitochondrial fractions are given for the two different LC columns used in Tables S1–S4. To enable comparison of values, we normalised raw ion intensities to those of an internal standard. As possible internal standards, we explored both taurine and glutathione, which are both abundant polar metabolites that are relatively evenly distributed between the mitochondrial matrix and the cytosol [12, 13]. Both are readily detected in both tissues and following LC on both columns used. While normalisation to either internal standard worked well in our hands, we settled on the use of taurine because in some cases the levels of glutathione may be altered by oxidation or alkylation [13]. The taurine‐normalised values for all metabolites are shown in Tables S5–S8.

### Rapid cooling and homogenisation stabilised mitochondrial metabolite amounts

**Fig. 4:**
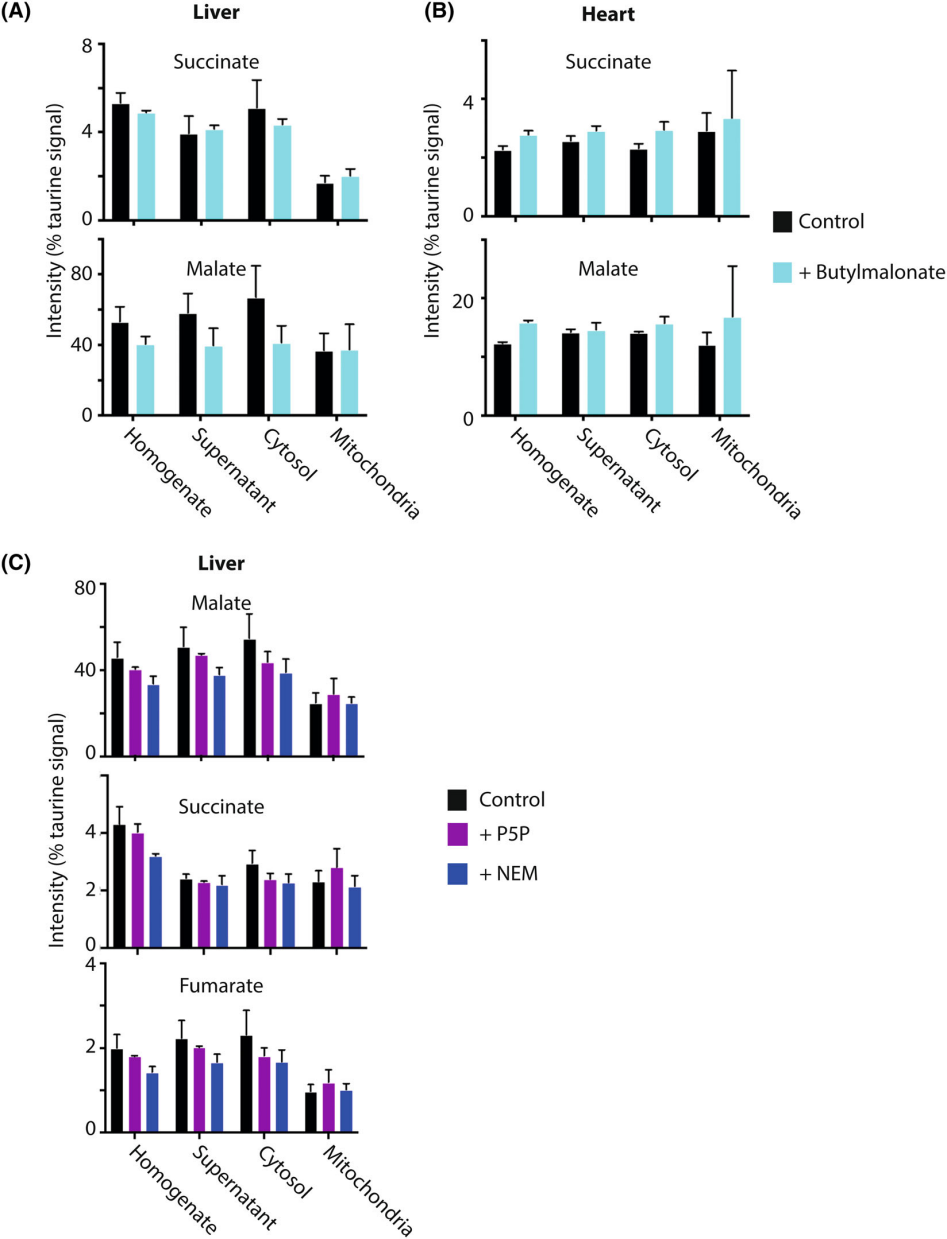
Effect of mitochondrial transport inhibitors on mitochondrial metabolite distribution. Mouse heart (A) and liver (B) tissues were rapidly processed as described in Fig. 1, ±500 μm butylmalonate and the taurine‐normalised levels of succinate and malate determined in the tissue homogenate, initial supernatant, cytosol and mitochondrial fractions. Data are mean ± range for control samples (*n* = 2) and mean ± SEM for butylmalonate samples (*n* = 3). (C) Mouse livers were rapidly processed as described in Fig. 1, in the presence or absence of 500 μm *N*‐ethylmaleimide (NEM) or 200 μm pyridoxal‐5‐phosphate (P5P) and the taurine‐normalised levels of succinate, malate and fumarate determined in the tissue homogenate, initial supernatant, cytosol and mitochondrial fractions. Data are means ± SEM, *n* = 3. Data were analysed by 2‐way ANOVA and no significant differences were found.

We next wanted to assess how effective were the rapid cooling and homogenisation of the tissues at preventing *ex vivo* changes in mitochondrial metabolite levels or distribution. To see whether there was any loss of mitochondrial metabolites upon tissue homogenisation, we assessed the effect of the mitochondrial dicarboxylate carrier (DIC) inhibitor butylmalonate on the distribution of its substrates, succinate and malate [14]. To do this, butylmalonate was added to the homogenate and the substrate distribution was compared with homogenate without butylmalonate (Fig. 4A,B). This showed that in both heart and liver mitochondria, there was no effect of butylmalonate. Next, we assessed the effect of *N*‐ethylmaleimide (NEM) which inhibits multiple mitochondrial transporters by alkylation of key cysteine residues [15] and of pyridoxal‐5‐phosphate (P5P), which affects lysine residues in transport proteins [16]. Addition of NEM or P5P prior to homogenisation showed no change in malate, succinate or fumarate (Fig. 4C). As fumarate is not transported across the mitochondrial inner membrane, its distribution gives an indication of the variation in these measurements. Thus, once the tissues are rapidly cooled, diluted and homogenised, the metabolite levels and distribution are stable. So, the levels assessed likely reflect those *in vivo* under aerobic conditions.

### Mitochondrial and cytosolic metabolite compartmentation within the normal heart

**Fig. 5:**
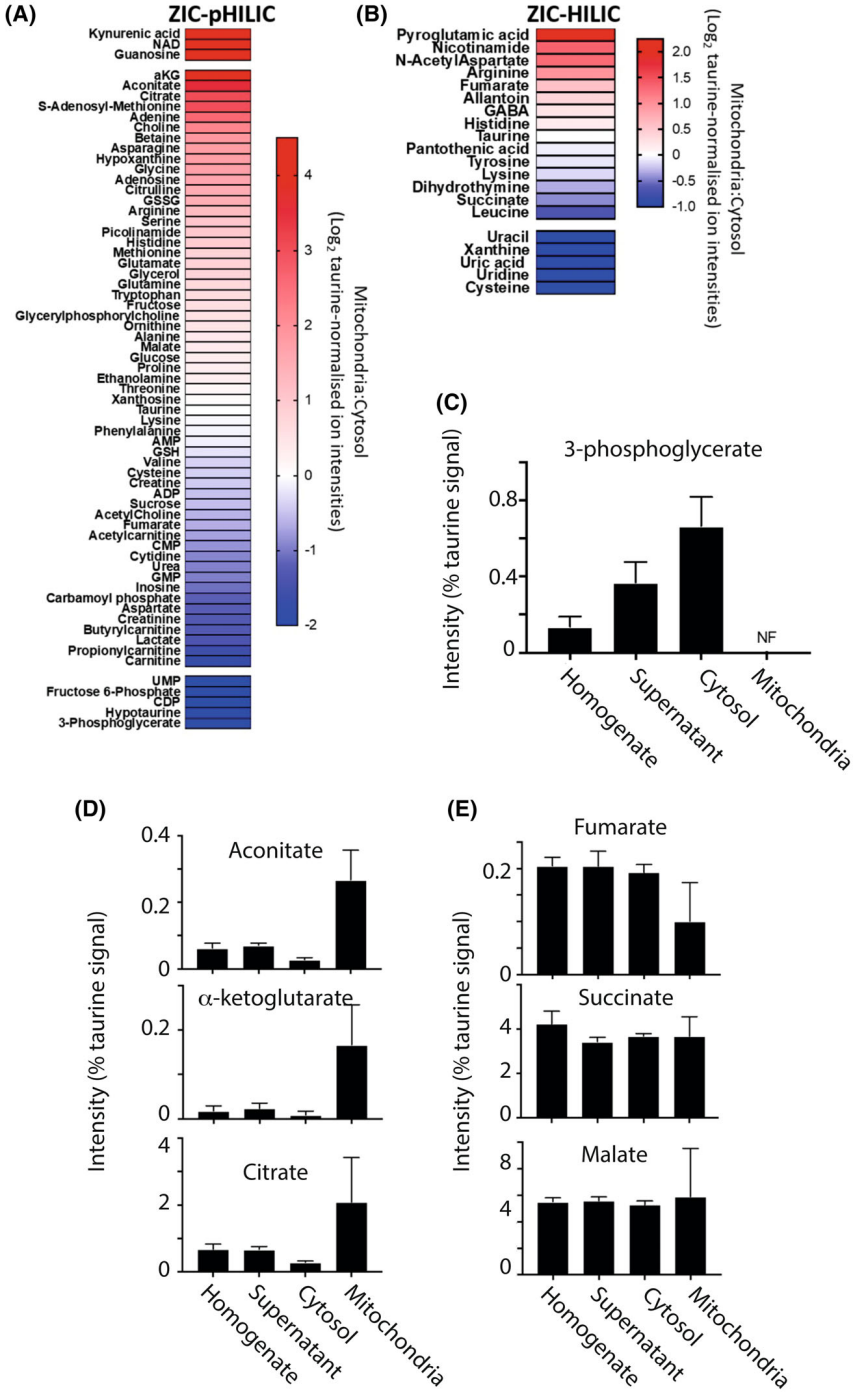
Metabolite distribution between heart mitochondria and cytosol. Metabolites from the control heart data set were normalised to taurine (Tables S5 and S6). For A and B, data are presented as Log_2_ of the ratio of mitochondrial : cytosolic levels normalised to taurine ion intensity, arranged in order of accumulation in the mitochondria. Data are average of six biological replicates. Metabolites which were assigned an adjusted zero value, due to no detection in the cytosolic or mitochondrial fraction, are placed above (cytosolic adjusted zero) or below (mitochondrial adjusted zero) the gaps in the heat map. (A) Metabolites analysed on the ZIC‐pHILIC column. (B) Metabolites analysed on the ZIC‐HILIC. (C) The distribution of glycolytic intermediates normalised to taurine in control mouse heart homogenates, supernatant, cytosol and mitochondria. Data are from a ZIC‐pHILIC column and are mean ± SEM of six biological replicates. (D, E) The distribution of TCA cycle intermediates normalised to taurine in control mouse heart homogenate, supernatant, cytosol and mitochondria. Data are from a ZIC‐pHILIC column, except for succinate which is from a ZIC‐HILIC column. Data are mean ± SEM of six biological replicates. NF, not found.

We next assessed the distribution of selected metabolites between mitochondria and the cytosol within heart tissue under normal conditions. The relative distribution was measured as the enrichment of the metabolite in the mitochondrial fraction versus the level in the cytosol and is shown as heat maps (Fig. 5A,B). The heat maps are separated based on the LC column used. It can be seen from the heat map that a number of metabolites are primarily found in the mitochondria (red) or in the cytosol (blue). For example, the glycolytic intermediate 3‐phosphoglycerate along with lactate was found only or mainly in the cytosol, but not detected in the mitochondria (Fig. 5C). Some TCA metabolites were only found within the mitochondria, including aconitate, α‐ketoglutarate and citrate (Fig. 5D). Interestingly, malate, fumarate and succinate were equally distributed between the cytosol and mitochondria (Fig. 5E). Both fumarate and malate can be generated and consumed in both compartments, so their dual distribution is unsurprising. As malate is readily transported by the DIC, there is likely to be interplay between the two pools. In contrast, fumarate is not itself directly transported across the mitochondrial inner membrane so the connection between the two pools is indirect, possibly mediated *via* malate transport across the inner membrane and the activity of malate dehydrogenase and fumarate hydratase in the two compartments connecting malate, oxaloacetate and fumarate pools. The situation with succinate is more interesting as it is only generated within mitochondria and then must be exported. This suggests that succinate is routinely exported from mitochondria to the cytosol *via* the DIC.

### Mitochondrial and cytosolic metabolite compartmentation within the normal liver

**Fig. 6:**
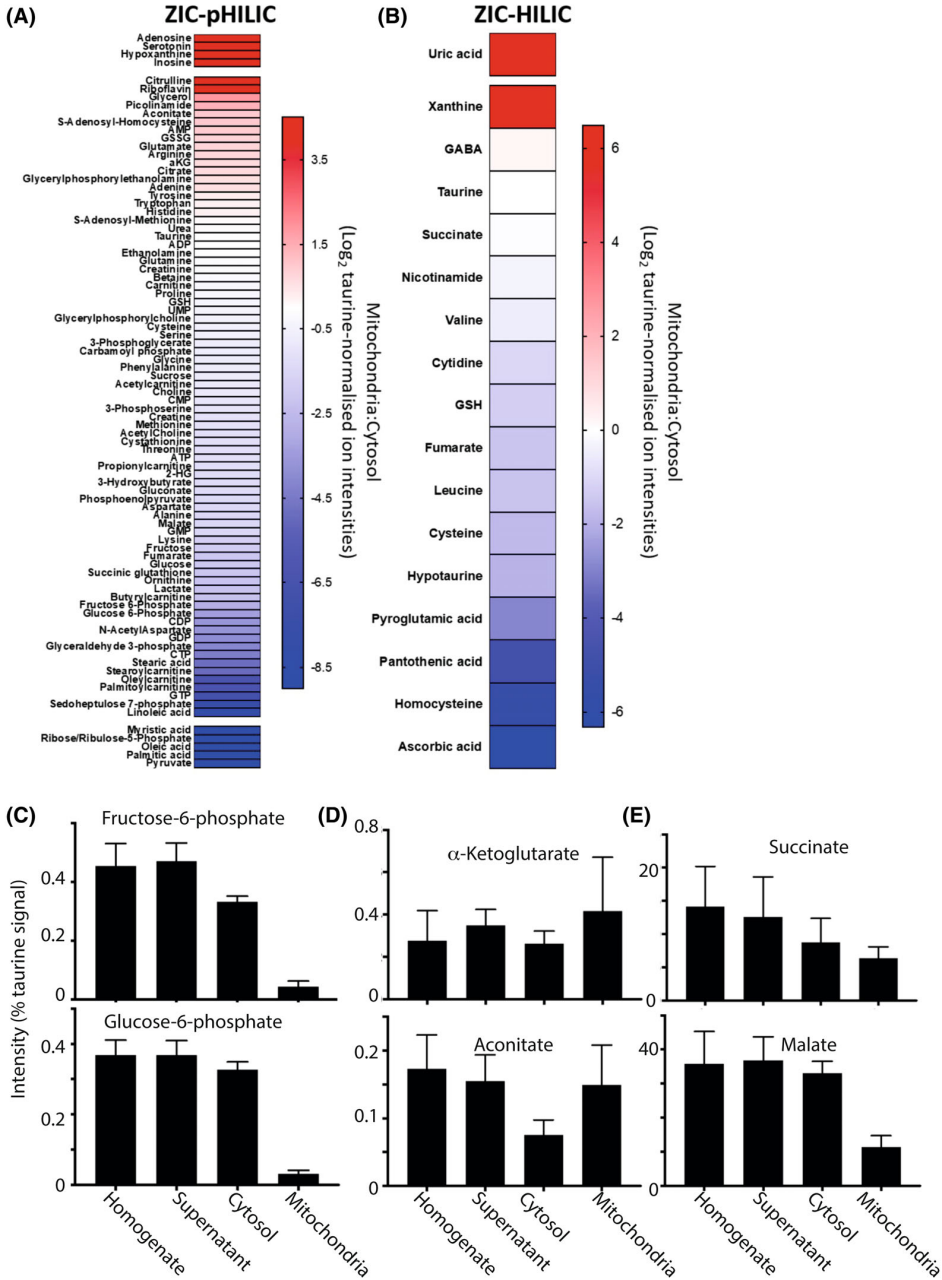
Metabolite distribution between liver mitochondria and cytosol. Metabolites from the control liver data set were normalised to taurine (Tables S7 and S8). For A and B, data are presented as Log_2_ of the ratio of mitochondrial:cytosolic levels normalised to taurine ion intensity, arranged in order of accumulation in the mitochondria. Data are average of six biological replicates. Metabolites that were assigned an adjusted zero value, due to no detection in the cytosolic or mitochondrial fraction, are placed above (cytosolic adjusted zero) or below (mitochondrial adjusted zero) the gaps in the heat map. (A) Metabolites analysed on the ZIC‐pHILIC column. (B) Metabolites analysed on the ZIC‐HILIC. (C) The distribution of the glycolytic intermediates normalised to taurine, in control mouse liver homogenate, supernatant, cytosol and mitochondria. Data are from ZIC‐pHILIC column and is mean ± SEM of six biological replicates. (D, E) the distribution of the TCA cycle intermediates normalised to taurine in control mouse liver homogenate, supernatant, cytosol and mitochondria. Data are from a ZIC‐pHILIC column, except for succinate which is from a ZIC‐HILIC column. Data are mean ± SEM of six biological replicates. NF, not found.

We next assessed the distribution of selected metabolites between mitochondria and the cytosol within liver tissue under normal conditions. The relative distribution was measured as the enrichment of the metabolite in the mitochondrial fraction versus the level in the cytosol and is shown as heat maps (Fig. 6A,B). The heat maps are separated based on the LC column used. It can be seen from the heat map that a number of metabolites are primarily found in the mitochondria (red) or in the cytosol (blue). For example, lactate and pyruvate, the pentose phosphate pathway intermediates ribose/ribulose‐5‐phosphate and sedoheptulose‐7‐phosphate, the glycolytic intermediates glyceraldehyde‐3‐phosphate, glucose‐6‐phosphate and fructose‐6‐phosphate were found only or largely in the cytosol (Fig. 6C). As expected, given the quite different functions of the two organs, their overall complements of metabolites were distinct, but our focus here was on their intracellular distribution. In contrast to the heart, some TCA metabolites that were only found within the mitochondria in the heart were found in both compartments in the liver, including aconitate and α‐ketoglutarate (Fig. 6D). As with the heart, malate and succinate were relatively equally present in both the cytosol and mitochondria (Fig. 6E). The greater presence of TCA cycle intermediates in the cytosol of the liver compared to the heart likely reflects the fact that liver export ‘acetyl’ units as citrate for fatty acid synthesis, whereas the heart does not express the tricarboxylate carrier needed for this.

### Mitochondrial and cytosolic metabolite compartmentation following warm ischaemia

**Fig. 7:**
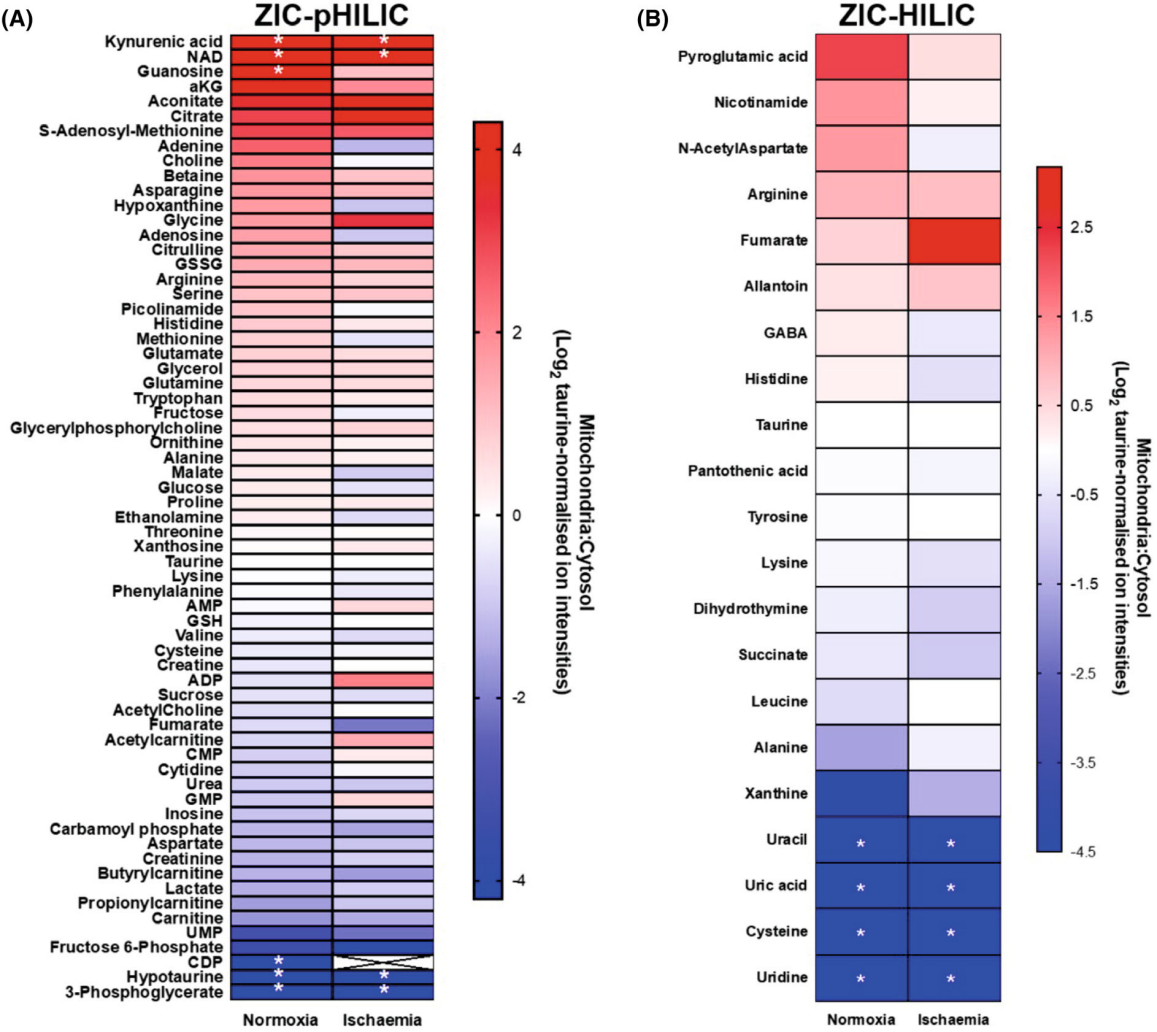
Metabolite enrichment in mouse heart mitochondria after 30 min warm ischaemia compared to that during normoxia. Metabolites from the control and ischaemic heart data sets are presented as Log_2_ of the ratio of mitochondrial : cytosolic levels normalised to taurine ion intensity, arranged in order of accumulation in the mitochondria. (A) Metabolites analysed on the ZIC‐pHILIC column. (B) Metabolites analysed on the ZIC‐HILIC column. *Metabolites that were not detected in the cytosolic (red) or mitochondrial (blue) fractions and were assigned an adjusted zero value. Crosses: blank values in both fractions. Data are average of six biological replicates.

**Fig. 8:**
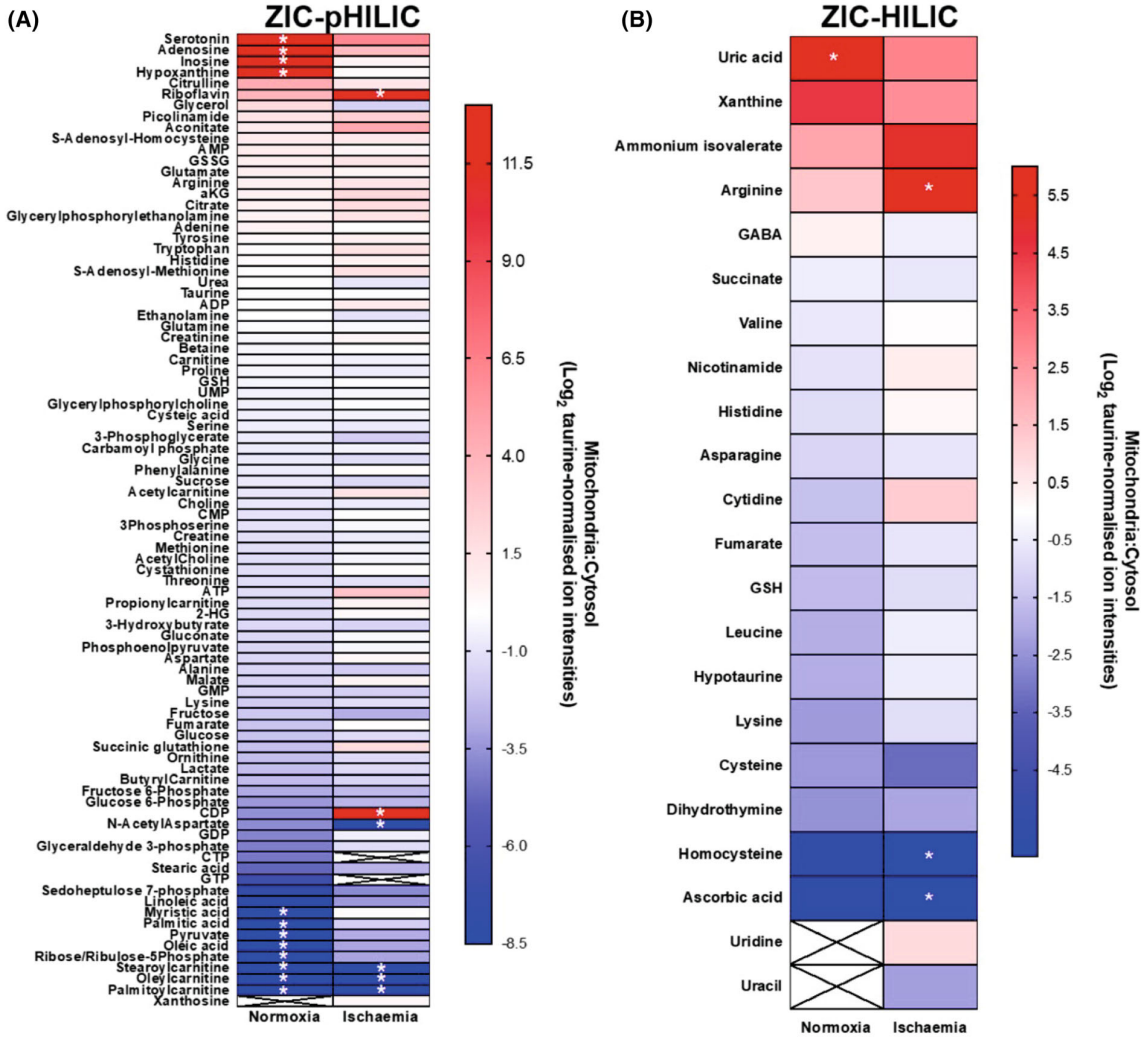
Metabolite enrichment in mouse liver mitochondria after 30 min warm ischaemia compared to that during normoxia. Metabolites from the control and ischaemic liver data sets are presented as Log_2_ of the ratio of mitochondrial:cytosolic levels normalised to taurine ion intensity, arranged in order of accumulation in the mitochondria. (A) Metabolites analysed on the ZIC‐pHILIC column. (B) Metabolites analysed on the ZIC‐HILIC column. *Metabolites that were not detected in the cytosolic (red) or mitochondrial (blue) fractions and were assigned an adjusted zero value. Crosses: blank values in both fractions. Data are average of three biological replicates.

A period of warm ischaemia (WI) dramatically alters metabolism, particularly those pathways associated with adenine nucleotide breakdown and succinate metabolism [17]. Therefore, as a proof of concept, to see if the rapid tissue fractionation procedures could be used to assess changes in metabolite distribution following a significant metabolic perturbation, we analysed mouse heart (Fig. 7) or liver (Fig. 8) after 30 min WI. To do this, we culled the mice and then left the tissues within the mice at 37 °C to expose them to WI and then determined the mitochondrial/cytosol distribution of metabolites as before and analysed and compared these with normoxia. As can be seen in Figs 7 and 8, for many metabolites the mitochondrial/cytosol distribution was altered markedly on going from normoxia to WI, thus. While the mechanisms and significance of these changes are beyond the scope of this work, it does illustrate that our rapid fractionation procedure can be applied to interrogate the effects of factors that alter metabolism.

## Conclusion

The distribution of metabolites between the mitochondria and cytosol *in vivo* is a key variable that must be assessed to help understand metabolic processes in signalling and pathology. To assist in this here, we have developed a rapid fractionation procedure that enables us to infer this distribution *in vivo*. This approach should prove useful to many researchers investigating the roles of metabolites in signalling and pathology *in vivo*, particularly where rapid redistributions of metabolites are being explored, such as during ischaemia‐reperfusion injury. Here we have focussed on liver and heart, but these procedures can easily be applied to other metabolically important tissues, such as brain and kidney. However, it will be important to optimise conditions for each organ, which was done here. Furthermore, the heart and liver are relatively homogeneous compared to organs such as the brain which have many different cell types with altered pools of mitochondrial metabolites.

## Author contributions

AMJ and MPM designed the study. FMA performed the experiments and analysed the data, with assistance from AVG, AMJ and CV. ASHC and CF performed the metabolomics and analysed the data. MPM wrote the manuscript which all authors read, edited and revised.

## Supporting information


**Fig. S1.** Western blots of mitochondria to assess contamination by other organelles.

**Table S1.** Control and 30 min WI heart metabolite ion intensities, ZIC‐pHILIC column.
**Table S2.** Control and 30 min WI heart metabolite ion intensities, ZIC‐HILIC column.
**Table S3.** Control and 30 min WI liver metabolite ion intensities, ZIC‐pHILIC column.
**Table S4.** Control and 30 min WI liver metabolite ion intensities, ZIC‐HILIC column.
**Table S5.** Control and 30 min WI heart metabolite taurine normalised ion intensities, ZIC‐pHILIC column.
**Table S6.** Control and 30 min WI heart metabolite taurine normalised ion intensities, ZIC‐HILIC column.
**Table S7.** Control and 30 min WI liver metabolite taurine normalised ion intensities, ZIC‐pHILIC column.
**Table S8.** Control and 30 min WI liver metabolite taurine normalised ion intensities, ZIC‐HILIC column.

## Data Availability

The data that support the findings of this study are available in the Supporting Information section of this manuscript.
