# Supplementary material for: Rapid fractionation of mitochondria from mouse liver and heart reveals in vivo metabolite compartmentation
Source: FEBS Lett. 2022 Oct 27;597(2):246–61. doi: 10.1002/1873-3468.14511 (PMC7614208; doi:10.1002/1873-3468.14511)
Supplement: Supplementary file 1 — Fig. S1. Western blots of mitochondria to assess contamination by other organelles. [file FEB2-597-246-s002.pdf]

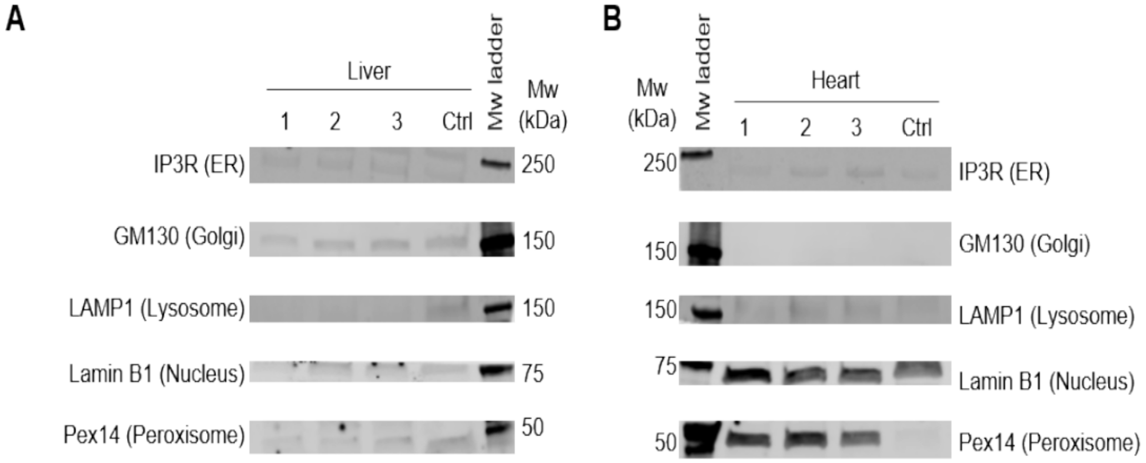

Figure S1. Western blot of cellular organelle contamination in rapidly isolated mouse heart and liver mitochondria. **A**, Liver mitochondria. **B**, Heart mitochondria. The following marker proteins were blotted to detect organelle contamination: IP3R (Endoplasmic reticulum); GM130 (Golgi apparatus); LAMP1 (Lysosome); Lamin B1 (Nucleus) and Pex14 (Peroxisome). Three separate preparations of rapidly isolated mitochondria (Lanes 1 - 3) and control mitochondria isolated by conventional method were separated by SDS-PAGE, transferred to PVDF membrane, cut in sections and probed with the indicated antibodies.
