# Supplementary material for: Rapid fractionation of mitochondria from mouse liver and heart reveals in vivo metabolite compartmentation
Source: FEBS Lett. 2022 Oct 27;597(2):246–61. doi: 10.1002/1873-3468.14511 (PMC7614208; doi:10.1002/1873-3468.14511)
Supplement: Supplementary file 2 — Table S1. Control and 30 min WI heart metabolite ion intensities, ZIC‐pHILIC column. Table S2. Control and 30 min WI heart metabolite ion intensities, ZIC‐HILIC column. Table S3. Control and 30 min WI liver metabolite ion intensities, ZIC‐pHILIC column. Table S4. Control and 30 min WI liver metabolite ion intensities, ZIC‐HILIC column. Table S5. Control and 30 min WI heart metabolite taurine normalised ion intensities, ZIC‐pHILIC column. Table S6. Control and 30 min WI heart metabolite taurine normalised ion intensities, ZIC‐HILIC column. Table S7. Control and 30 min WI liver metabolite taurine normalised ion intensities, ZIC‐pHILIC column. Table S8. Control and 30 min WI liver metabolite taurine normalised ion intensities, ZIC‐HILIC column. [file FEB2-597-246-s001.docx]

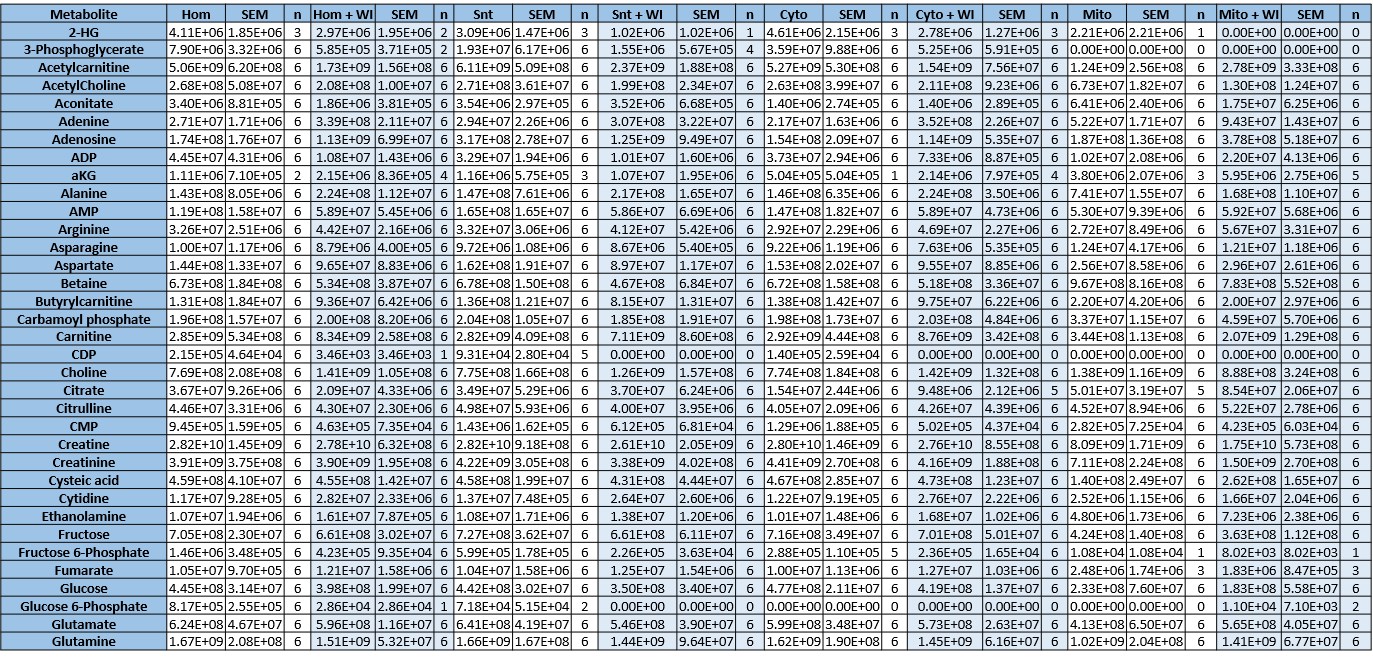


**Table S1** Control and 30 min WI heart metabolite ion intensities, ZIC-pHILIC column. 1 of 2.


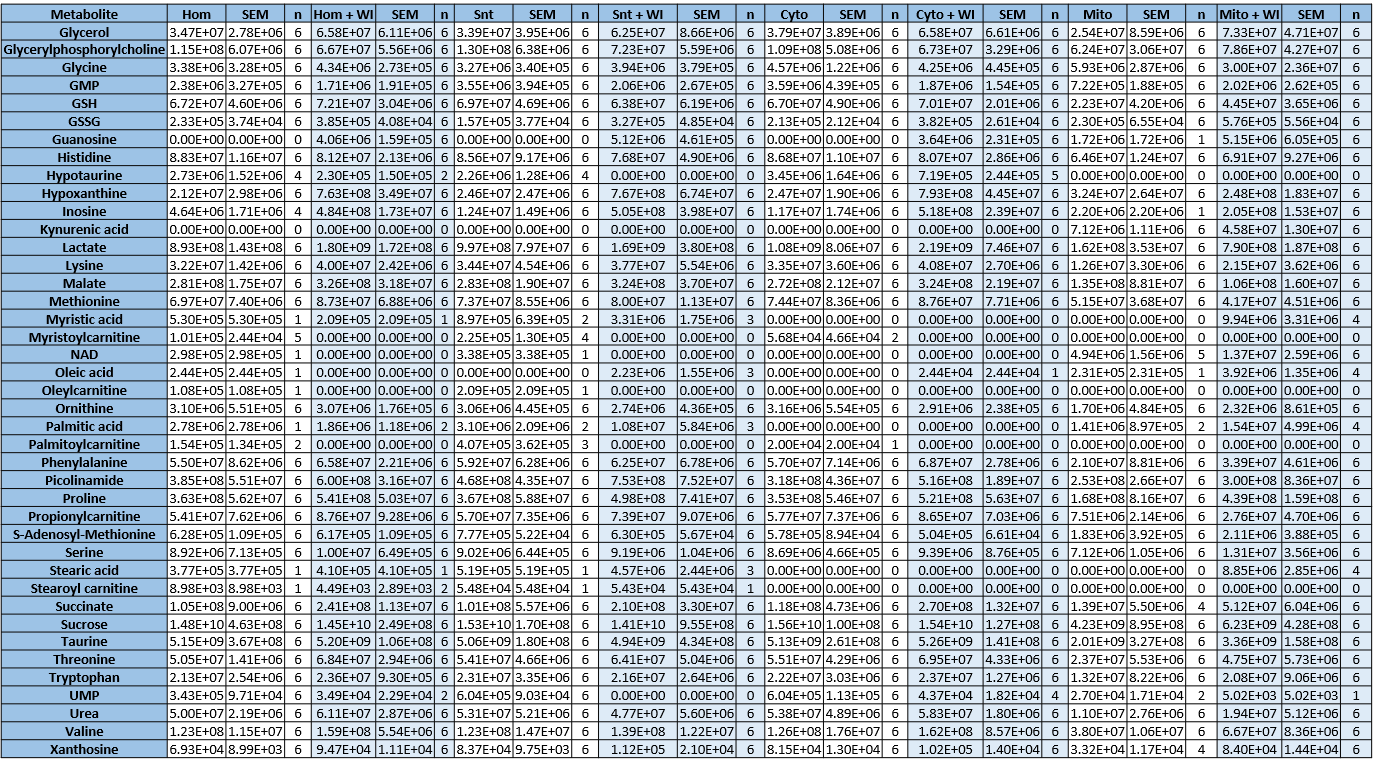


**Table S1** Control and 30 min WI heart metabolite ion intensities, ZIC-pHILIC column. 2 of 2.


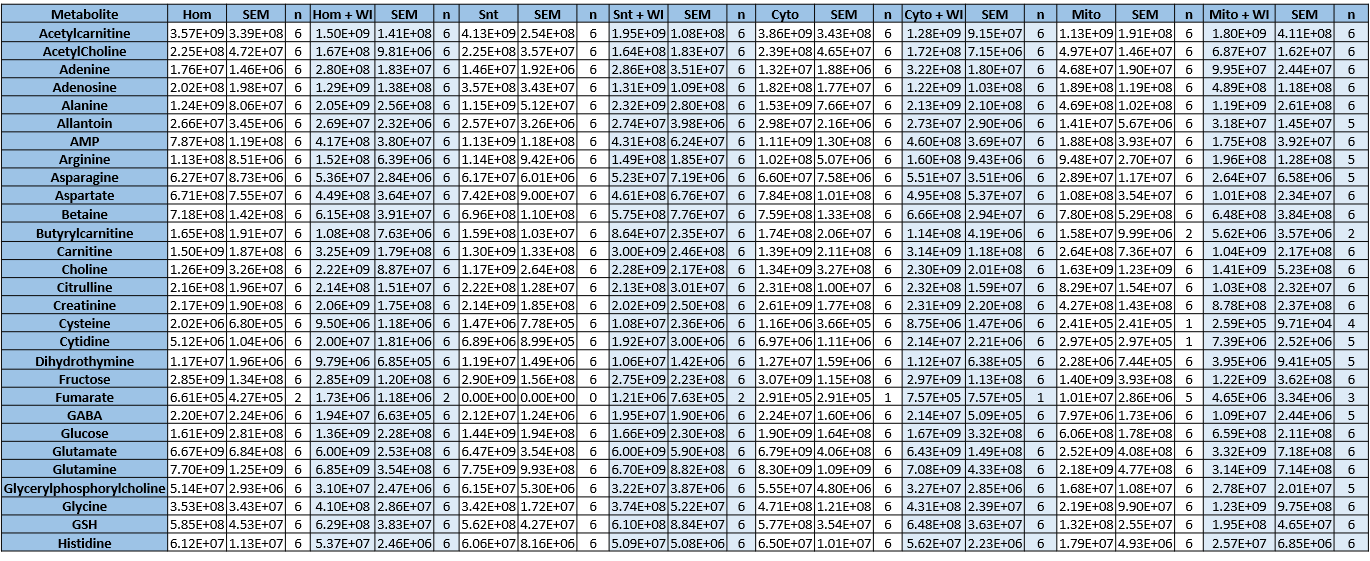
 **Table S2** Control and 30 min WI heart metabolite ion intensities, ZIC-HILIC column. 1 of 2.


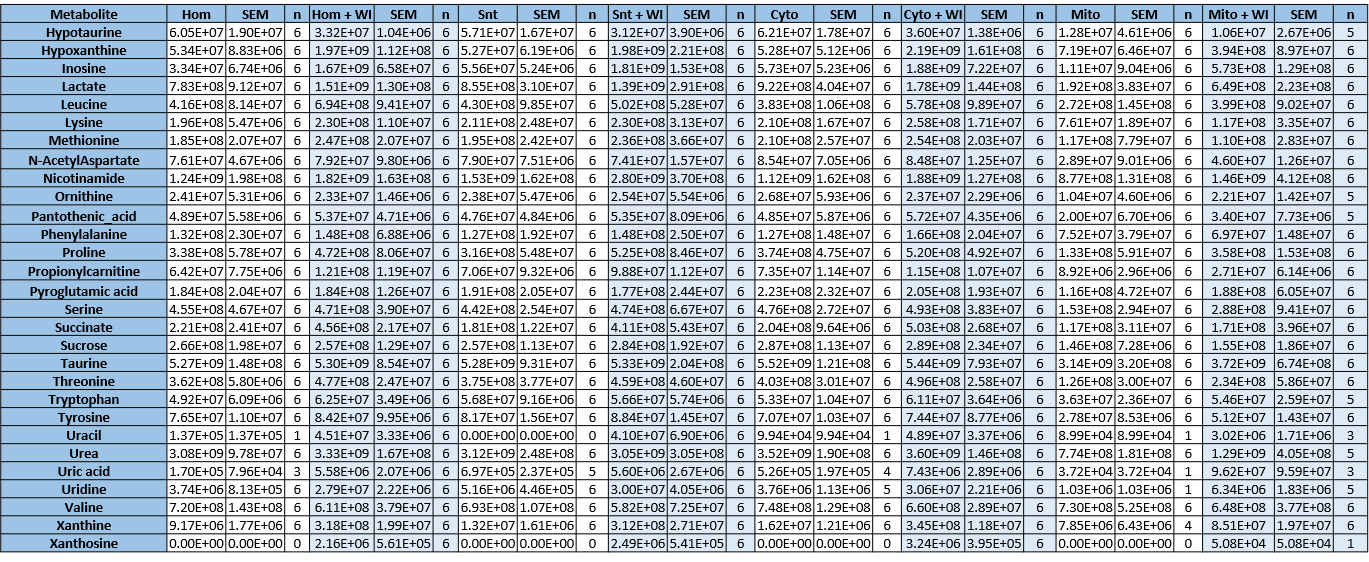
 **Table S2** Control and 30 min WI heart metabolite ion intensities, ZIC-HILIC column. 2 of 2.


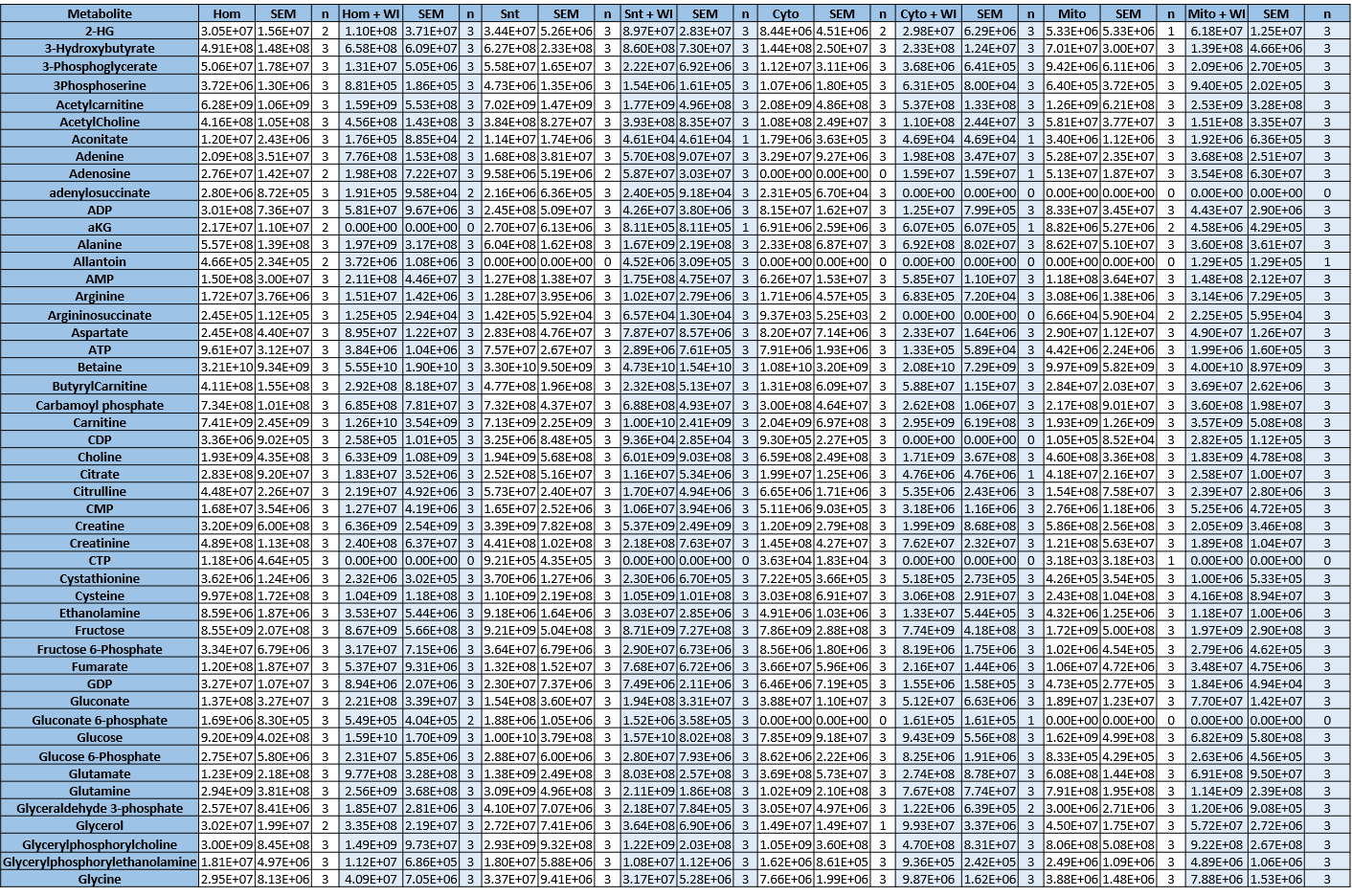


**Table S3** Control and 30 min WI liver metabolite ion intensities, ZIC-pHILIC column. 1 of 2.


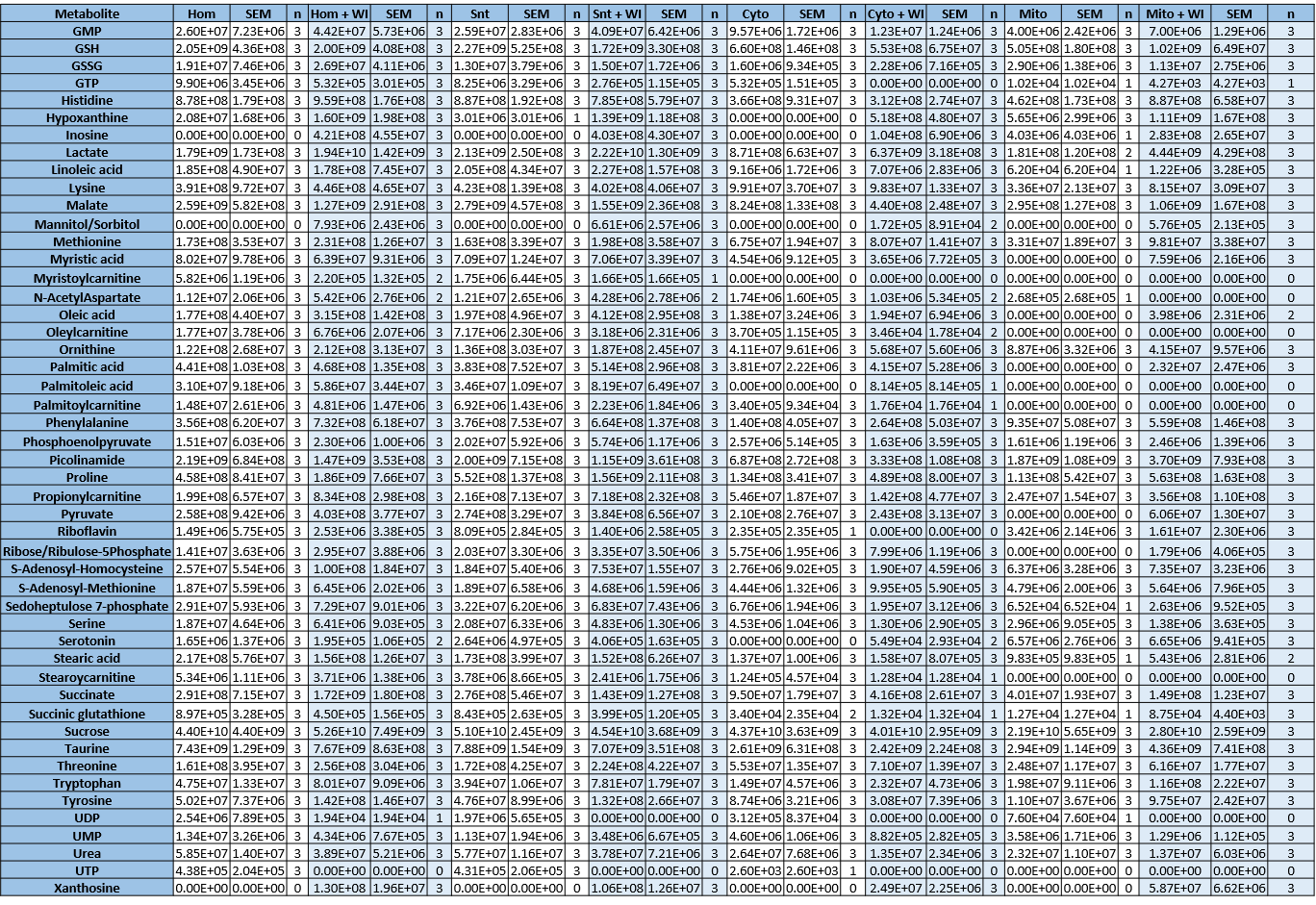


**Table S3** Control and 30 min WI liver metabolite ion intensities, ZIC-pHILIC column. 2 of 2.


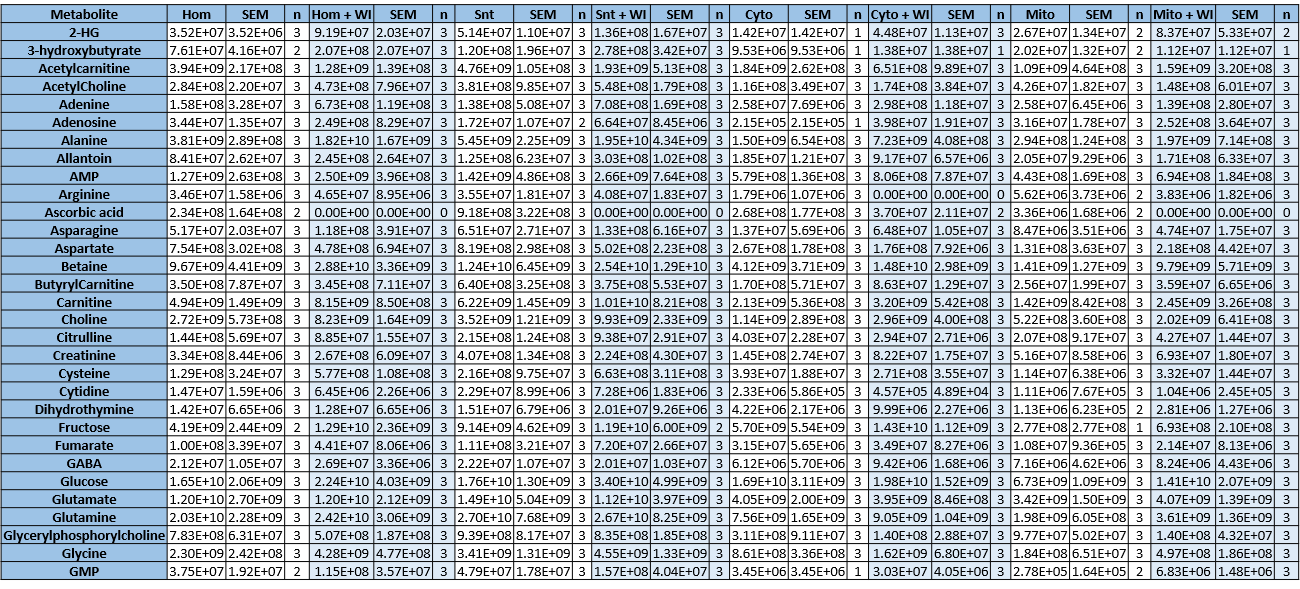


**Table S4** Control and 30 min WI liver metabolite ion intensities, ZIC-HILIC column. 1 of 2.


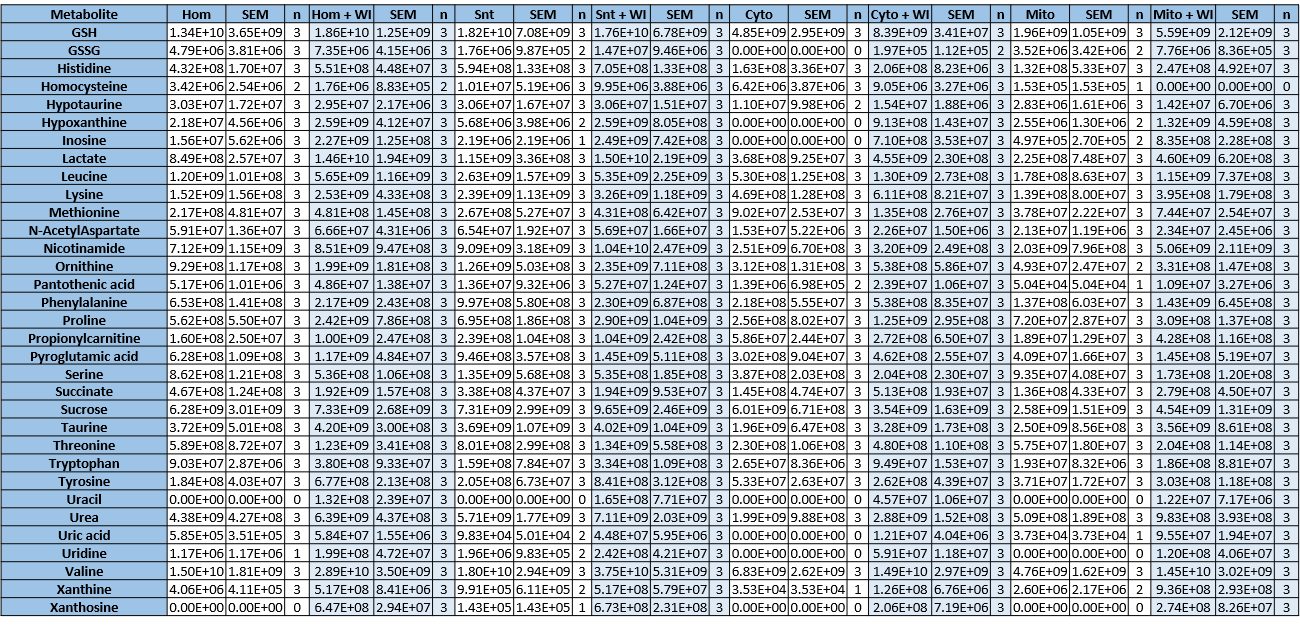


**Tablbe S4** Control and 30 min WI liver metabolite ion intensities, ZIC-HILIC column. 2 of 2.


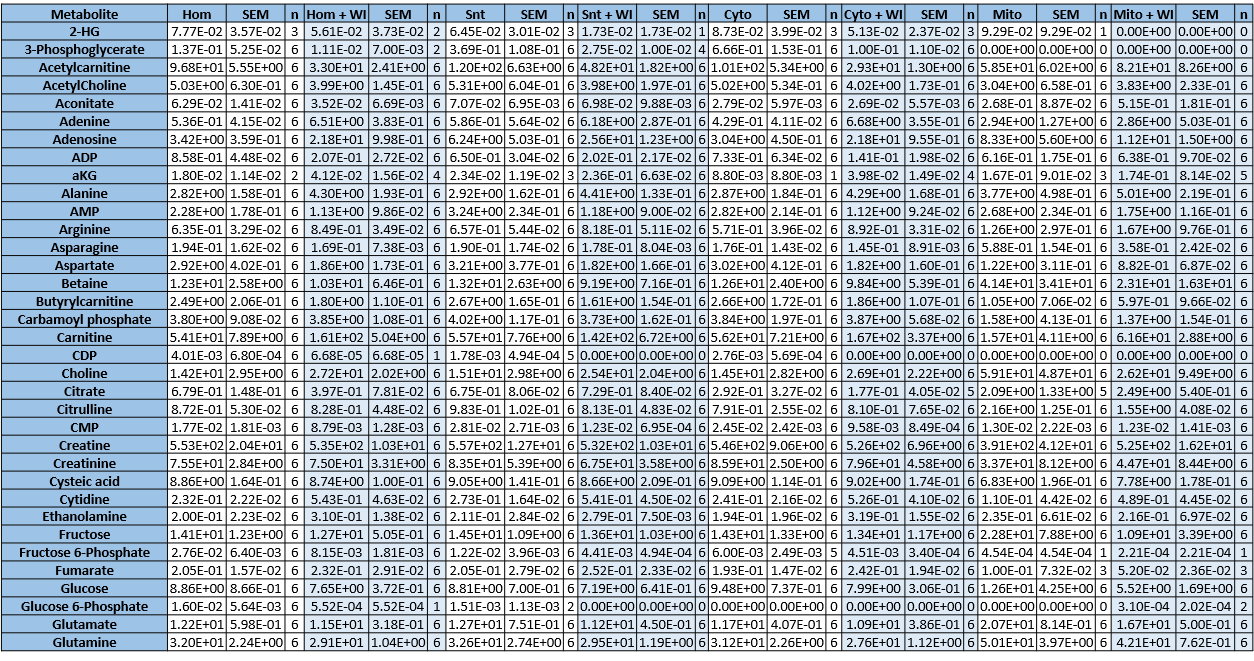


**Table S5** Control and 30 min WI heart taurine-normalised ion intensities, ZIC-pHILIC column. 1 of 2.


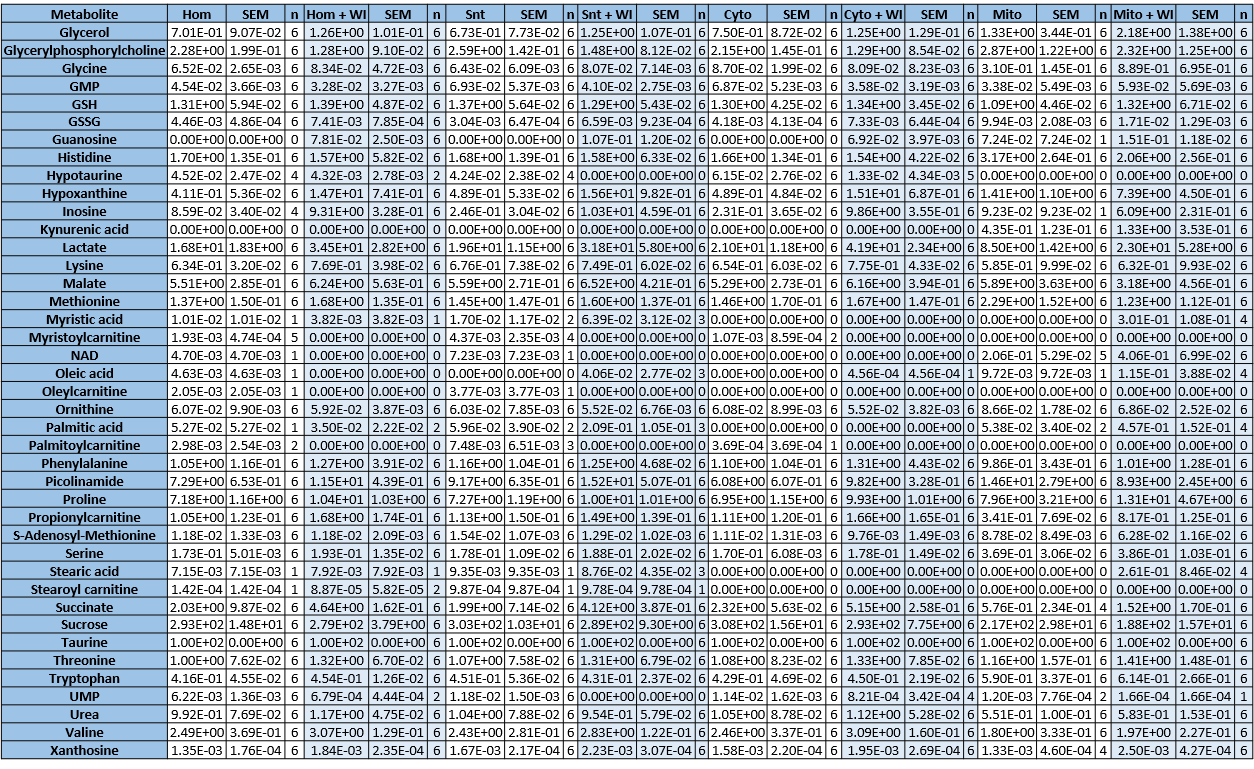


**Table S5** Control and 30 min WI heart taurine-normalised ion intensities, ZIC-pHILIC column. 2 of 2.


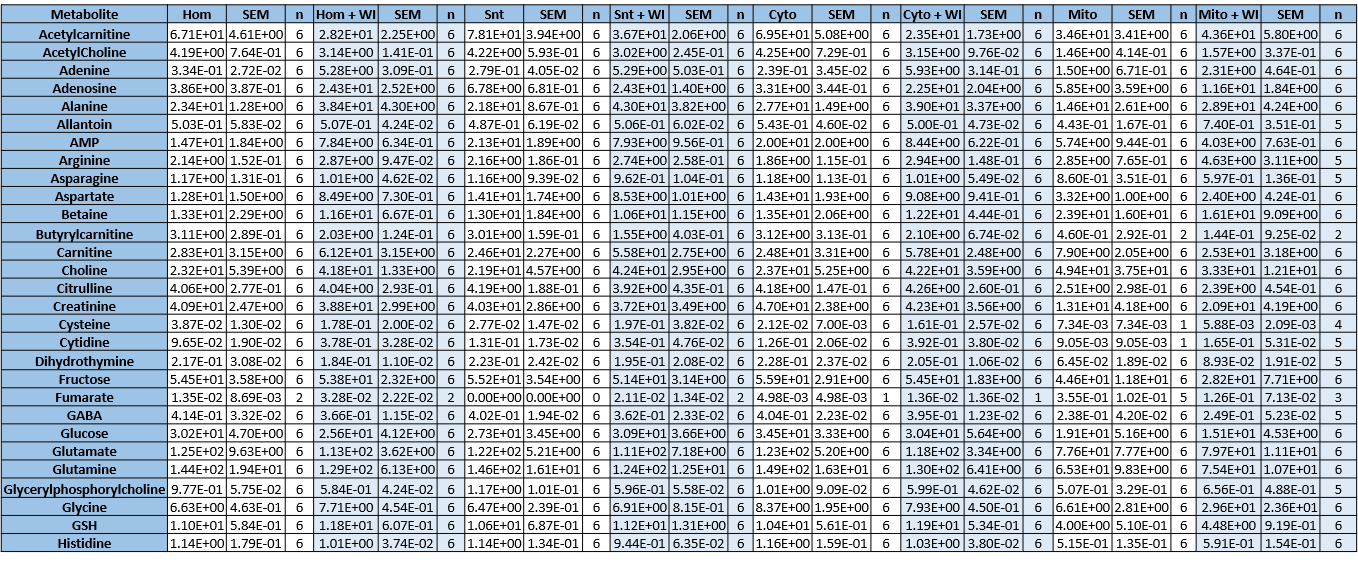


**Table S6** Control and 30 min WI heart taurine-normalised ion intensities, ZIC-HILIC column. 1 of 2.


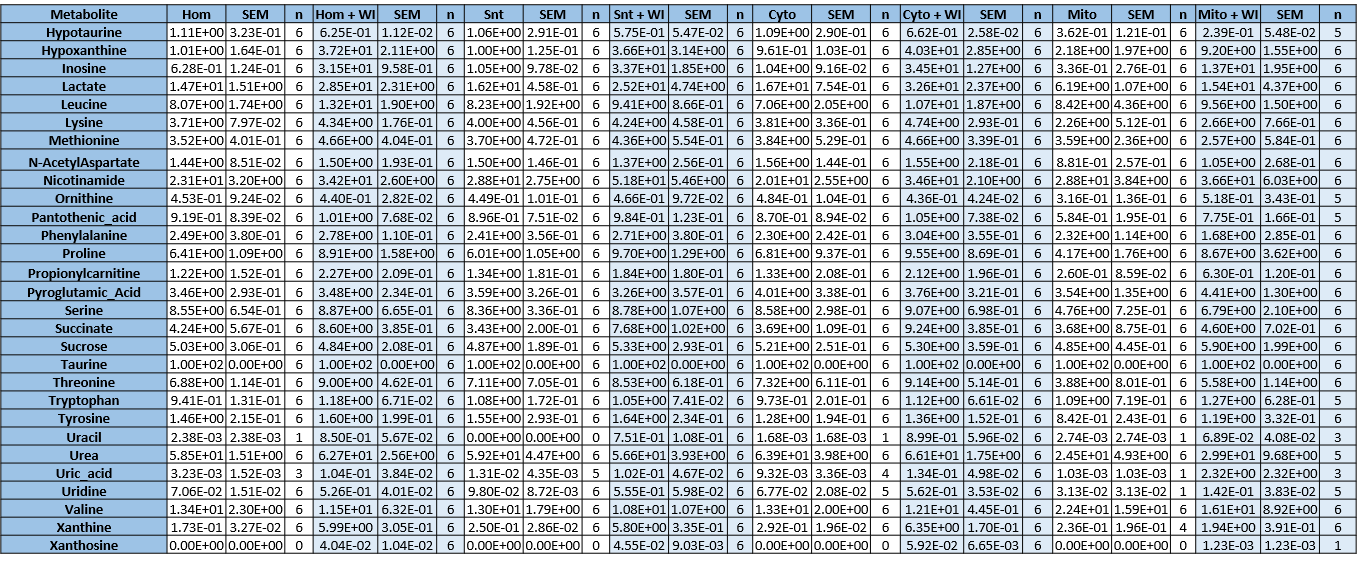


**Table S6** Control and 30 min WI heart taurine-normalised ion intensities, ZIC-HILIC column. 2 of 2.


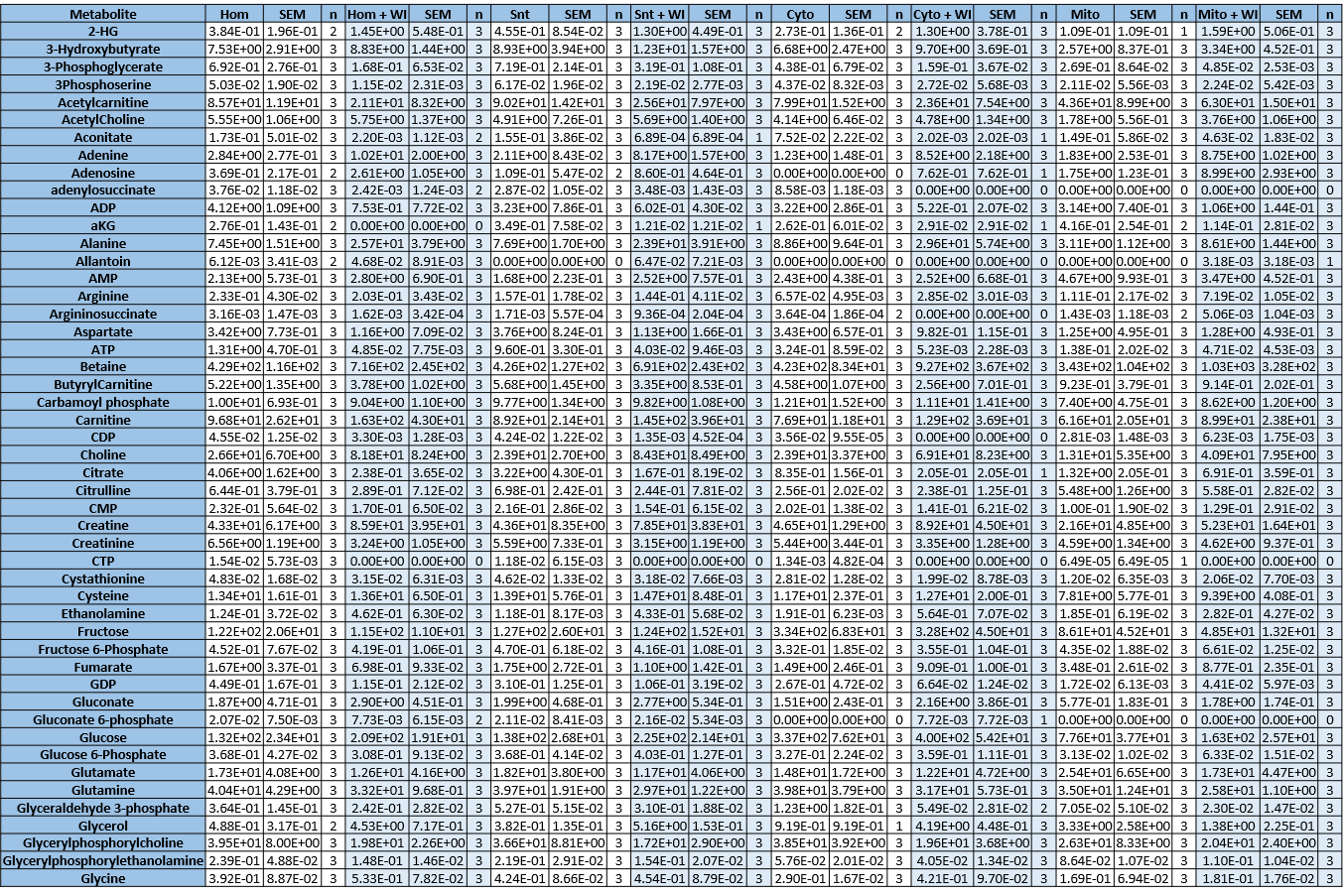


**Table S7** Control and 30 min WI liver taurine-normalised ion intensities, ZIC-pHILIC column. 1 of 2.


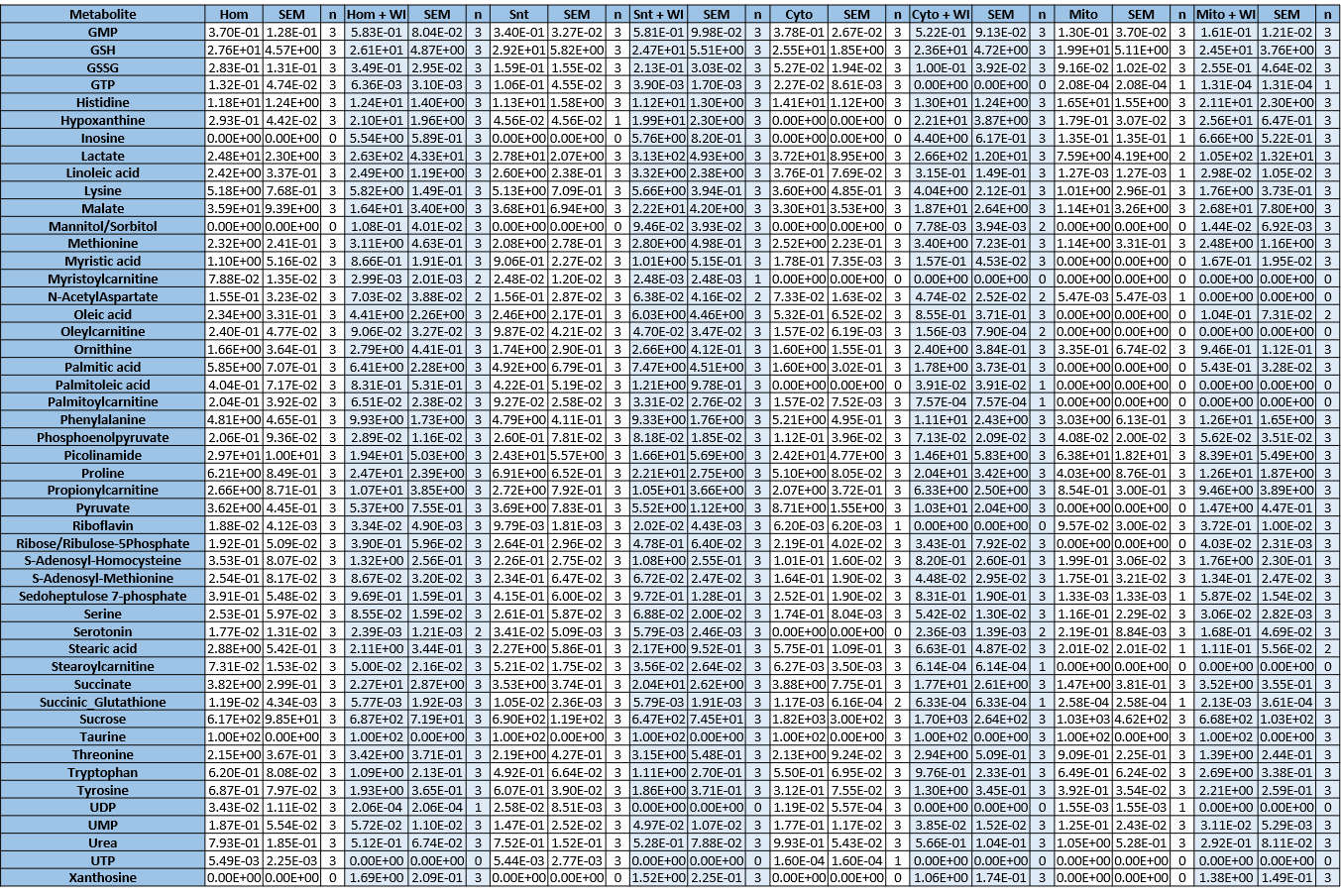


**Table S7** Control and 30 min WI liver taurine-normalised ion intensities, ZIC-pHILIC column. 2 of 2.


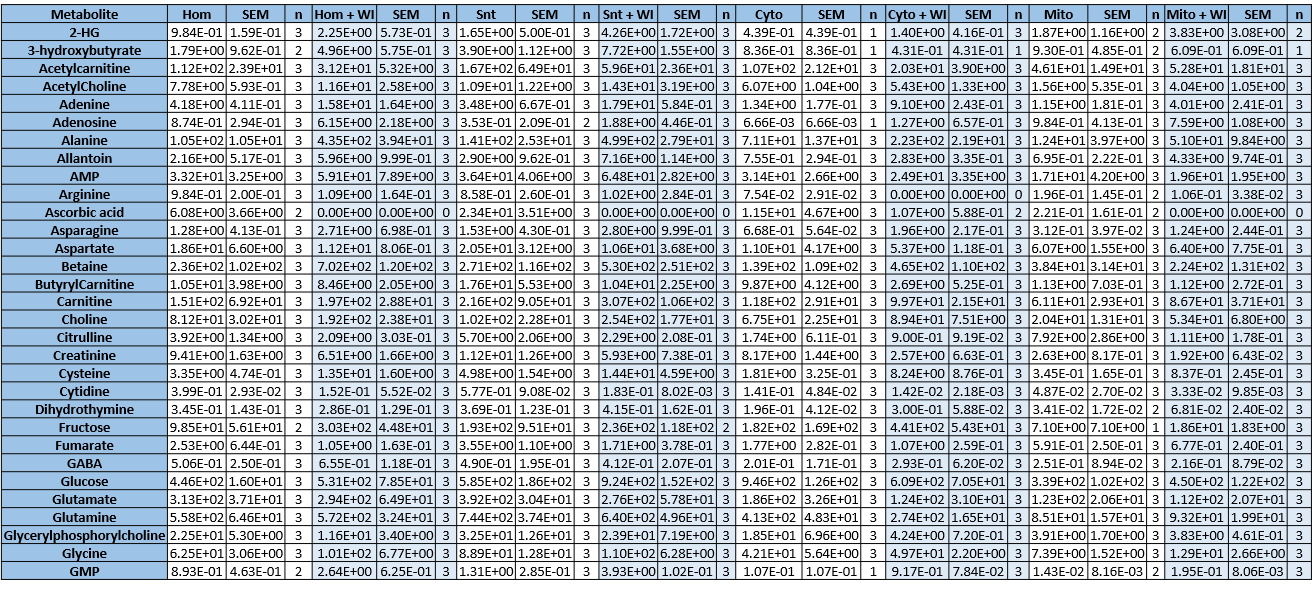


**Table S8** Control and 30 min WI liver taurine-normalised ion intensities, ZIC-HILIC column. 1 of 2.


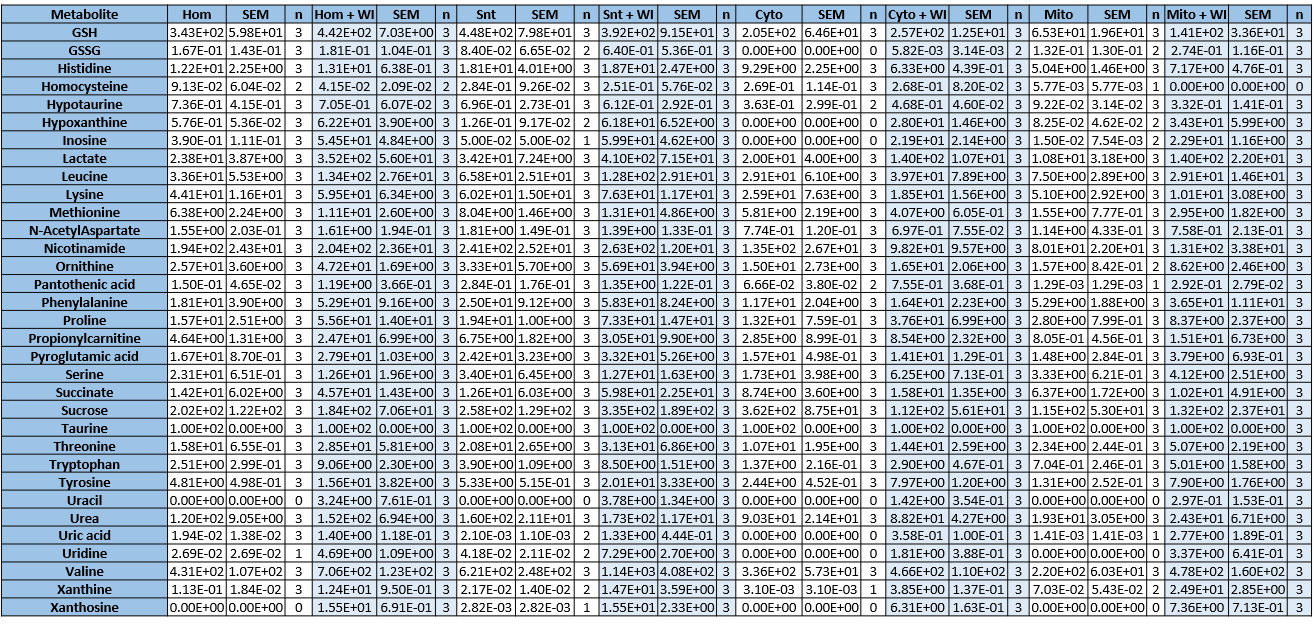


**Table S8** Control and 30 min WI liver taurine-normalised ion intensities, ZIC-HILIC column. 2 of 2.
